# Specification of claustro-amygdalar and palaeocortical neurons and circuits

**DOI:** 10.1038/s41586-024-08361-5

**Published:** 2025-01-15

**Authors:** Navjot Kaur, Rothem Kovner, Forrest O. Gulden, Mihovil Pletikos, David Andrijevic, Tianjia Zhu, John Silbereis, Mikihito Shibata, Akemi Shibata, Yuting Liu, Shaojie Ma, Nikkita Salla, Xabier de Martin, Thomas S. Klarić, Megan Burke, Daniel Franjic, Hyesun Cho, Matthew Yuen, Ipsita Chatterjee, Paula Soric, Devippriya Esakkimuthu, Markus Moser, Gabriel Santpere, Yann S. Mineur, Kartik Pattabiraman, Marina R. Picciotto, Hao Huang, Nenad Sestan

**Affiliations:** 1 Department of Neuroscience, Yale School of Medicine, New Haven, CT USA; 2 Department of Radiology, Children’s Hospital of Philadelphia, Philadelphia, PA USA; 3 Department of Bioengineering, School of Engineering and Applied Science, University of Pennsylvania, Philadelphia, PA USA; 4 Institute of Neuroscience, CAS Center for Excellence in Brain Science and Intelligence Technology, University of Chinese Academy of Sciences, Chinese Academy of Sciences, Shanghai, China; 5 Yale Child Study Center, New Haven, CT USA; 6 Neurogenomics Group, Hospital del Mar Research Institute, PRBB, Barcelona, Spain; 7 Institute of Experimental Hematology, School of Medicine, Techical University of Munich, Munich, Germany; 8 Department of Psychiatry, New Haven, CT USA; 9 Wu Tsai Institute, Yale University, New Haven, CT USA; 10 Interdepartmental Neuroscience Program, Yale University School of Medicine, New Haven, CT USA; 11 Department of Radiology, Perelman School of Medicine, University of Pennsylvania, Philadelphia, PA USA; 12 Department of Comparative Medicine, Yale University, New Haven, CT USA; 13 Department of Genetics, Yale University, New Haven, CT USA; 14 Kavli Institute for Neuroscience, Yale University, New Haven, CT USA; 15 Program in Cellular Neuroscience, Neurodegeneration and Repair, Yale University, New Haven, CT USA

**Keywords:** Developmental biology, Neuronal development, Evolution

## Abstract

The ventrolateral pallial (VLp) excitatory neurons in the claustro-amygdalar complex and piriform cortex (PIR; which forms part of the palaeocortex) form reciprocal connections with the prefrontal cortex (PFC), integrating cognitive and sensory information that results in adaptive behaviours^1–5^. Early-life disruptions in these circuits are linked to neuropsychiatric disorders^4–8^, highlighting the importance of understanding their development. Here we reveal that the transcription factors SOX4, SOX11 and TFAP2D have a pivotal role in the development, identity and PFC connectivity of these excitatory neurons. The absence of SOX4 and SOX11 in post-mitotic excitatory neurons results in a marked reduction in the size of the basolateral amygdala complex (BLC), claustrum (CLA) and PIR. These transcription factors control BLC formation through direct regulation of *Tfap2d* expression. Cross-species analyses, including in humans, identified conserved *Tfap2d* expression in developing excitatory neurons of BLC, CLA, PIR and the associated transitional areas of the frontal, insular and temporal cortex. Although the loss and haploinsufficiency of *Tfap2d* yield similar alterations in learned threat-response behaviours, differences emerge in the phenotypes at different *Tfap2d* dosages, particularly in terms of changes observed in BLC size and BLC–PFC connectivity. This underscores the importance of *Tfap2d* dosage in orchestrating developmental shifts in BLC–PFC connectivity and behavioural modifications that resemble symptoms of neuropsychiatric disorders. Together, these findings reveal key elements of a conserved gene regulatory network that shapes the development and function of crucial VLp excitatory neurons and their PFC connectivity and offer insights into their evolution and alterations in neuropsychiatric disorders.

## Abstract (web summary)

A conserved gene regulatory network involving *SOX4*, *SOX11* and *TFAP2D* shapes the development of excitatory neurons in ventrolateral pallium and their connectivity with the prefrontal cortex.

## Main

During embryonic development, pallial–subpallial boundary (PSB) progenitors give rise to immature excitatory neurons, which migrate via the lateral cortical stream (LCS) towards VLp, into the BLC and PIR primordia^9–18^. Although the patterning and differentiation of these progenitors are well studied, the molecular mechanism governing the specification and connectivity of excitatory neurons within these VLp structures has remained largely unexplored. Here we identify an evolutionarily divergent yet parallel gene regulatory network comprising SOX4, SOX11 and TFAP2D, which orchestrates the post-mitotic, cell-autonomous specification of BLC and PIR excitatory neuron features, including their projections to the PFC, and their functional contributions.

## SOX4 and SOX11 regulate VLp development

**Fig. 1:**
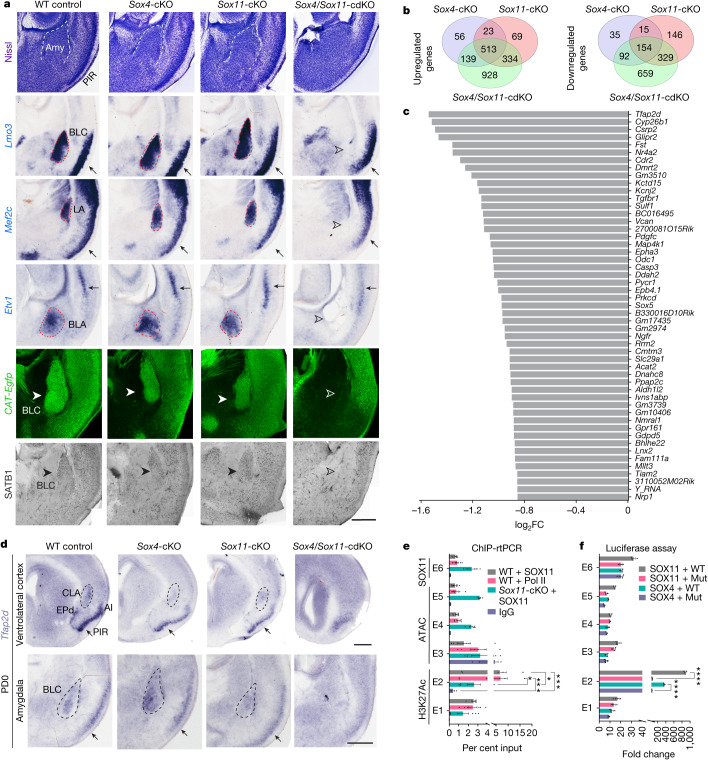
Regulation of VLp development and *Tfap2d* expression by SOX4 and SOX11. **a**, Coronal sections from VLp of mouse brains from wild-type (WT), *Sox4*-cKO, *Sox11*-cKO and *Sox4/Sox11*-cdKO mice. Nissl staining highlights the BLC. In situ hybridization for *Lmo3*,* Mef2c* and *Etv1* marks BLC, LA and BLA, whereas GFP and SATB1 immunostaining labels BLC. White and black filled arrowheads highlight normal phenotypes and open arrowheads highlight defective phenotypes. Scale bar, 200 μm. **b**, Intersection of differentially expressed genes in *Sox4*-cKO, *Sox11*-cKO and *Sox4/Sox11*-cdKO cortex and amygdala compared with controls (*n* = 3 per genotype). **c**, Top 50 genes with decreased expression in *Sox4/Sox11*-cdKO samples over *Sox4*-cKO, *Sox11-*cKO and control samples. FC, fold change. **d**, In situ hybridization for *Tfap2d* in the VLp of control, *Sox4*-cKO, *Sox11*-cKO and *Sox4/Sox11*-cdKO brains. Scale bars, 500 μm. **e**, Quantitative PCR showing the percentage of input of six potential *Tfap2d* enhancers (E1–E6) after ChIP on PCD15.5 wild-type and *Sox11*-cKO cortices, targeting IgG, SOX11 or Pol II. Two-way ANOVA with multiple comparisons and Tukey’s correction. Data are mean ± s.e.m. WT + SOX11 immunoprecipitation versus IgG: *P* = 0.0006; WT + Pol II immunoprecipitation versus IgG: *P* = 0.0003; WT + SOX11 immunoprecipitation versus *Sox11*-cKO: *P* = 0.033; WT + Pol II immunoprecipitation versus *Sox11*-cKO: *P* = 0.018. SOX11 and Pol II: *n* = 6; IgG: *n* = 3. **f**, Luciferase activity driven by the *Tfap2d* enhancers E1–E6 and mutants (Mut) for SOX4 and SOX11 binding sites in the presence SOX4 or SOX11 as measured by firefly luciferase/*Renilla* luciferase ratio. Data are normalized to enhancer activity in the absence of SOX4 or SOX11. Two-way ANOVA with Tukey’s correction. Data are mean ± s.e.m. *n* = 3 per condition. Detailed statistics are presented in Supplementary Table 6. AI, anterior insula; Amy, amygdala; EPd, EP, dorsal part. ****P < 0.0001. In **a**,**d**, slanted arrows point to PIR; horizontal arrows point to neocortex; and hollow arrowheads point to loss of BLC in *Sox4*/*Sox11*-cdKO.

**Extended Data Fig. 1:**
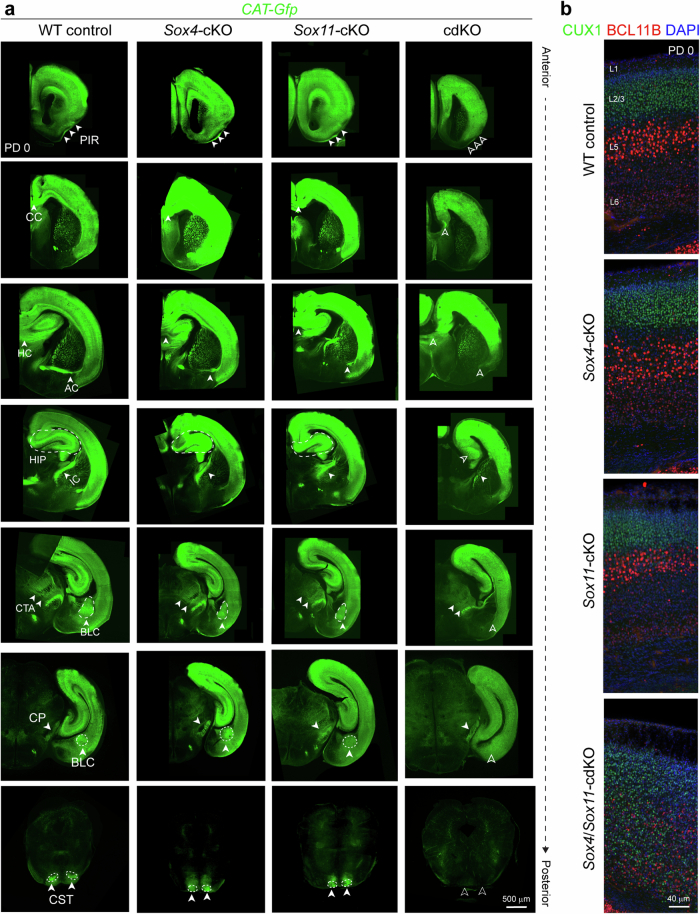
Loss of *Sox4* and *Sox11* disrupts laminar organization and axonal projections of cortical excitatory neurons. **a**) Serial sections of control, *Sox4*-cKO, *Sox11-*cKO and *Sox4; Sox11*-cdKO mice brains at PD 0. Filled arrows point to normal phenotype and open arrowhead point to defective phenotypes in the knockouts. **b**) Representative images illustrate immunostaining for CUX1 and BCL11B in the cerebral cortex, highlighting disrupted laminar organization and decreased density nuclei with pronounced BCL11B immunosignal in the *Sox4*; *Sox11*-cdKO brains, in contrast to control, *Sox4*-cKO, and *Sox11*-cKO samples. AC, anterior commissure; CC, corpus callosum; CP, cerebral peduncles; CST, corticospinal tract; CTA, corticothalamic axons; HC, hippocampal commissure; HIP, hippocampus; IC, internal capsule; L, layer.

**Extended Data Fig. 2:**
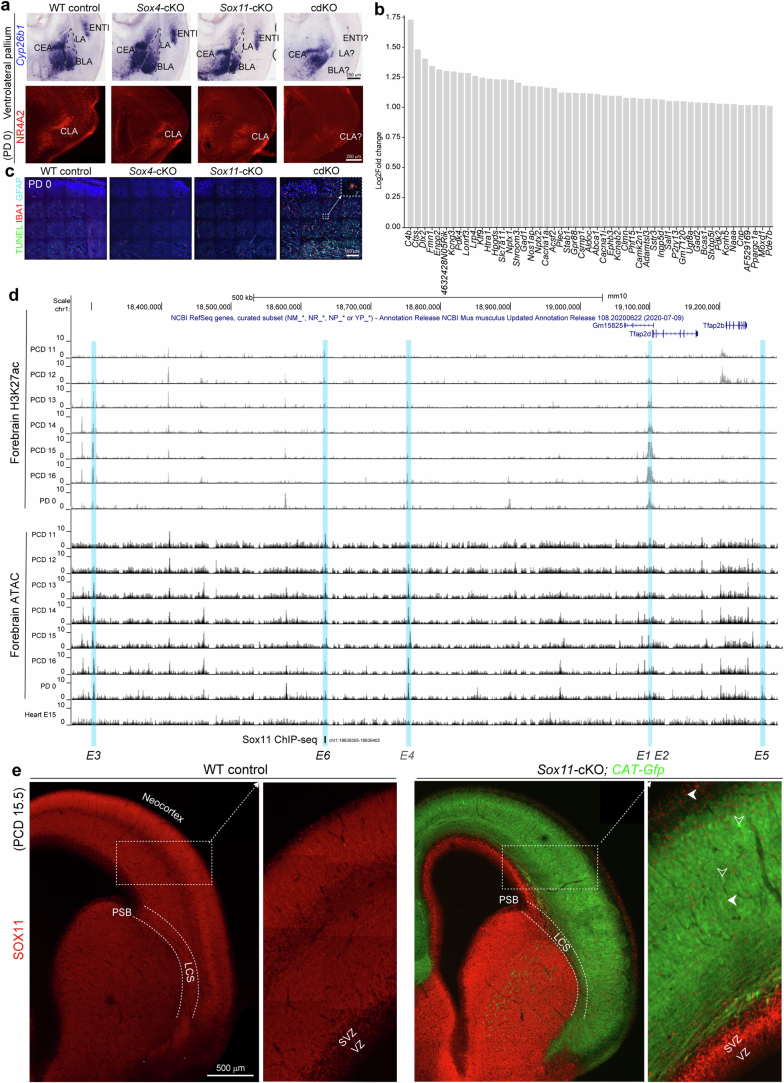
*Sox4* and *Sox11* orchestrate VLC formation by regulating *Tfap2d* expression for cell survival, and development of VLp. **a**) Coronal sections of the in situ hybridization for *Cyp26b1* (upper panel) and immunostaining for NR4A2 validating their downregulation in the ventrolateral cortical structures- including BLC, CLA, and ENTI in *Sox4*; *Sox11*-cdKO as compared to control, *Sox4*-cKO, and *Sox11*-cKO brains. In all genotypes, *Cyp26b1* expression is robust in the CEA, the GABAergic inhibitory center of amygdala. **b**) Bar graph showing top 50 genes that have increased in expression in *Sox4; Sox11*-cdKO samples as compared to *Sox4*-cKO, *Sox11*-cKO and control within cortex and amygdala at PD 0. **c**) Representative images showing the immunostaining for GFAP and IBA1 along with TUNEL assay to depict the cell damage, astrocytes and microglia invasion in the *Sox4; Sox11*-cdKO as compared to the control and *Sox4*-cKO, *Sox11*-cKO cortices at PD 0. CEA, central nucleus of the amygdala; CLA, claustrum; ENTI, entorhinal cortex; LA, lateral nucleus of the amygdala. **d**) Line graphs showing the forebrain H3K27ac^32^ and ATAC peaks^33^ and *SOX11* ChIP-seq sites^29^ from ages PCD 11-16, and PD 0 at the *Tfap2d* genomic locus ATAC peaks from heart (bottom tract) samples depict that the mechanism is forebrain specific. The blue boxes highlight putative enhancers (*E*) harboring predicted binding sites for *SOX11*, spanning from *E1* to *E6*, with ChIP-seq confirmation of binding specifically within *E6*. **e**) Validation of the anti-*SOX11* antibody involved examining coronal sections of PDC 15.5 brains. In WT control brains, *SOX11* immunostaining (red) was observed, while in the *SOX11*-cKO, expression from all GFP+ excitatory neurons was lost (indicated by open arrowheads). Filled arrowheads point to *SOX11* immunopositive nuclei within the cortical plate and in SVZ. ATAC; Assay for Transposase-Accessible Chromatin. SVZ, subventricular zone; VZ, ventricular zone; WT, wildtype.

In prior observations, we noted that depletion of the transcription factors SOX4 and SOX11 from *Emx1*-expressing cortical progenitors resulted in notable developmental defects in the cerebral cortex^19^. These defects included the loss of *Fezf2* expression, disruption laminar specification of excitatory neurons, and impaired corticospinal tract formation. Furthermore, SOX4 and SOX11 are expressed in immature cerebral excitatory neurons, yet their specific post-mitotic functions remain poorly understood beyond these phenotypes^19–21^. To investigate these functions, we bred *Neurod6-cre*;CAT-Gfp mice with *Sox4*^*fl/fl*^ and *Sox11*^*fl/fl*^ mice to selectively eliminate post-mitotic expression of both *Sox4* and *Sox11* from cerebral excitatory neurons (*Sox4/Sox11* conditional double knockout (cdKO)) while simultaneously marking these cells with green fluorescent protein (GFP). Given the observed redundancy in the function of SOX4 and SOX11^19,20^, *Sox4*-conditional knockout (cKO) and *Sox11-*cKO mice did not exhibit noticeable phenotypic defects compared with control littermates at postnatal day 0 (PD0) (Fig. 1a and Extended Data Figs. 1a,b and 2a,c). Similar to our previous observations^19^, brains of *Sox4/Sox11*-cdKO mice showed phenotypic defects in corticospinal tract and cortical layer formation, highlighting post-mitotic functions of SOX4 and SOX11 (Extended Data Fig. 1a,b). Further, in *Sox4/Sox11*-cdKO mice, we observed pronounced reduction of VLp size, encompassing the BLC, CLA and PIR (Fig. 1a and Extended Data Fig. 1a). Notably, these structures also express both *Sox4* and *Sox11* during development^19^. Nissl staining, in situ hybridization and immunostaining for markers outlining nuclei of the amygdala confirmed that *Sox4/Sox11*-cdKO mice almost entirely lacked BLC, which comprises the basolateral (BLA), lateral (LA) and basomedial (BMA) nuclei, at PD0 (Fig. 1a and Extended Data Fig. 2a). Specifically, *Lmo3* and SATB1 comprehensively delineated the entire BLC; *Etv1* and *Mef2c* distinctly marked BLA and LA, respectively; and *Cyp26b1* prominently labelled both the BLA and the central amygdala nuclei^18,19,22^. Additionally, NR4A2 (also known as NURR1) immunostaining revealed a reduction in the size of the endopiriform nucleus (EP) and CLA in *Sox4/Sox11*-cdKO brains (Extended Data Fig. 2a).

## SOX4 and SOX11 regulate *Tfap2d* expression in VLp

To investigate downstream genes regulated by SOX4 and SOX11, we performed bulk tissue RNA-sequencing (RNA-seq) analysis on pooled cerebral cortex and amygdala tissue obtained from PD0 in *Sox4/Sox11*-cdKO, *Sox4*-cKO and *Sox11*-cKO mice and control littermates. We identified 958 and 659 genes that were uniquely upregulated and downregulated, respectively, in the *Sox4/Sox11*-cdKO brains (Fig. 1b and Supplementary Tables 1 and 2). Because neuroinflammation-associated genes were upregulated in the *Sox4/Sox11*-cdKO brains, we next assessed whether deletion of *Sox4* or *Sox11*, or both led to increased microglia and astroglia invasion (Extended Data Fig. 2b and Supplementary Table 1). Increased labelling of the glial markers IBA1 and GFAP by immunofluorescence confirmed microglia and astroglia invasion, respectively (Extended Data Fig. 2c). In addition, TUNEL staining provided evidence for DNA fragmentation, suggesting cell damage (Extended Data Fig. 2c). These data corroborate previously reported functions of SOX4 and SOX11 in neuron differentiation and survival^20,21^.

**Fig. 2:**
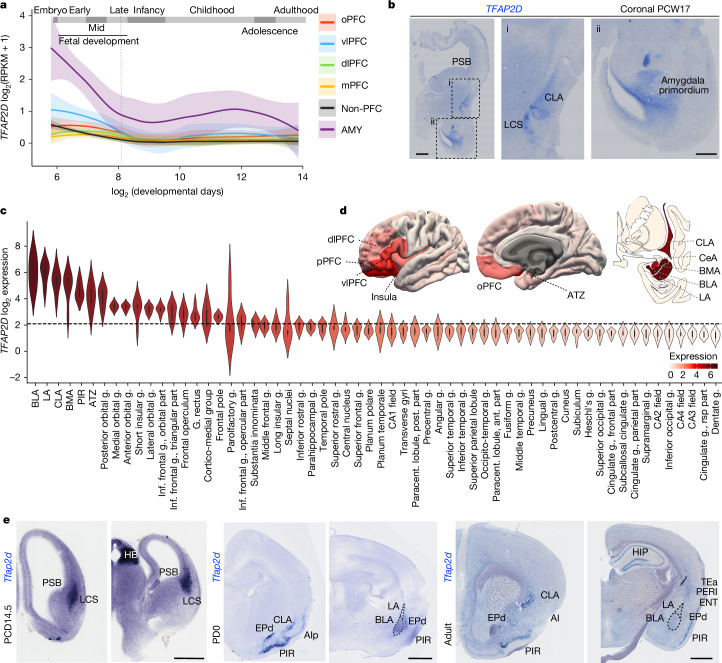
Conserved expression pattern of *TFAP2D* in human and mouse brain. **a**, Expression of *TFAP2D* across developmental ages in the human amygdala (AMY), orbital PFC (oPFC), ventral PFC (vPFC), dorsolateral PFC (dlPFC), medial PFC (mPFC) and non-PFC^28,29^. Data are mean ± s.e.m. **b**, Coronal section of a human brain at PCW17, showing the expression of *TFAP2D* in the LCS, CLA and amygdala primordium. Scale bars, 2 mm (left), 1 mm (right). **c**, *TFAP2D* expression pattern across frontal cortical regions, VLp and subcortical regions in adult human brain as derived from publicly available microarray data^31^. The dashed line represents the mean of *TFAP2D* expression across all regions represented here. Ant., anterior; g., gyrus; inf., inferior; paracent., paracentral; post., posterior; rsp., retrospinal. **d**, Human brain images showing *TFAP2D* expression gradients across the adult brain. Correspondence between the Desikan–Killiany atlas and the Allen Brain Atlas probes^51^ was used to visualize *TFAP2D* expression. Subcortical regions were visualized using data from http://atlas.brain-map.org. ATZ, amygdalohippocampal transition zone; CEA, central nucleus of the amygdala. **e**, In situ hybridization for *Tfap2d* in coronal sections of wild-type mice at PCD14.5, PD0 and in adult. Scale bars, 200 μm. AIp, AI, posterior part; ENTI, entorhinal cortex; HB, habenula; HIP, hippocampus; PERI, perirhinal cortex; pPFC, polar PFC.

Because the severe defects in the VLp were seen only in the *Sox4/Sox11*-cdKO mice, we focused on downregulated genes (Fig. 1b and Supplementary Table 2). Among these, many have been previously shown^23–27^ to be enriched in BLC, CLA or PIR, including *Cyp26b1*, *Glipr2*, *Fst* and *Nr4a2* (Fig. 1c and Extended Data Fig. 2a). The most downregulated gene in *Sox4/Sox11*-cdKO mice encodes the transcription factor TFAP2D (also known as AP-2 delta) (Fig. 1c and Supplementary Table 2), which was previously shown to be enriched in human and non-human primate amygdala^23,24,28,29^. Although TFAP2D has previously been implicated in development of dorsal midbrain^25^ and retina^26^, its role in development of amygdala or other VLp structures has not been studied. In situ hybridization and transcriptomic analysis revealed that *Tfap2d* exhibits increased expression in developing excitatory neurons in the BLC and neighbouring VLp structures, such as the PIR, CLA, EP anterior olfactory nucleus and anterior insula (Figs. 1d and 2e, Extended Data Fig. 5c and Supplementary Table 2). In accordance with the results of the RNA-seq analysis, expression of *Tfap2d* was almost absent in these structures of *Sox4/Sox11*-cdKO mice when assessed at PD0 (Fig. 1d).

We investigated SOX4 and SOX11 regulation of *Tfap2d* by integrating chromatin immunoprecipitation with sequencing (ChIP–seq) for H3K27ac^27^ and assay for transposase-accessible chromatin using sequencing (ATAC-seq)^30^ from the mouse forebrain (post-conception day (PCD) 11.5 to PD0), with predicted SOX-binding motifs and SOX11 ChIP–seq data^21^ (Extended Data Fig. 2d). Using this analysis, we identified six putative enhancers (E1–E6) associated with *Tfap2d* expression, which also showed increased accessibility starting at PCD13.5, which coincides with the migration of immature amygdalar excitatory neurons^13,18^. To validate these in silico findings, we conducted chromatin immunoprecipitation followed by real-time PCR (rtPCR) using a SOX11 antibody that we validated in *Sox11*-cKO brains (Extended Data Fig. 2e), and crosslinked chromatin from PCD16.5 wild-type and *Sox11*-cKO cortices and amygdalae. We could not identify a suitable SOX4 antibody for immunohistochemistry or ChIP assays. Our ChIP results revealed strong binding of SOX11 and RNA polymerase II (Pol II) to E2 (Fig. 1e), indicating its active involvement in SOX11-dependent *Tfap2d* transcription. Further, by performing luciferase reporter assays for all six enhancers (E1–E6) in *Neuro2a* cells, we show that SOX4 and SOX11 expression increased the luciferase activity of the E2 enhancer, whereas mutating putative SOX4 and SOX11 sites markedly reduced this activity (Fig. 1f). These results strongly suggest that SOX11 and SOX4 directly regulate *Tfap2d* via the E2 enhancer, highlighting their pivotal role in this regulatory mechanism. Consistent with these findings, *Sox4*, *Sox11* and *Tfap2d* are co-expressed in BLC excitatory neurons (Extended Data Figs. 4c,d,  7a–d and 8a–d). Collectively, these findings indicate that SOX11 directly regulates the expression of *Tfap2d* in the VLp.

**Extended Data Fig. 3:**
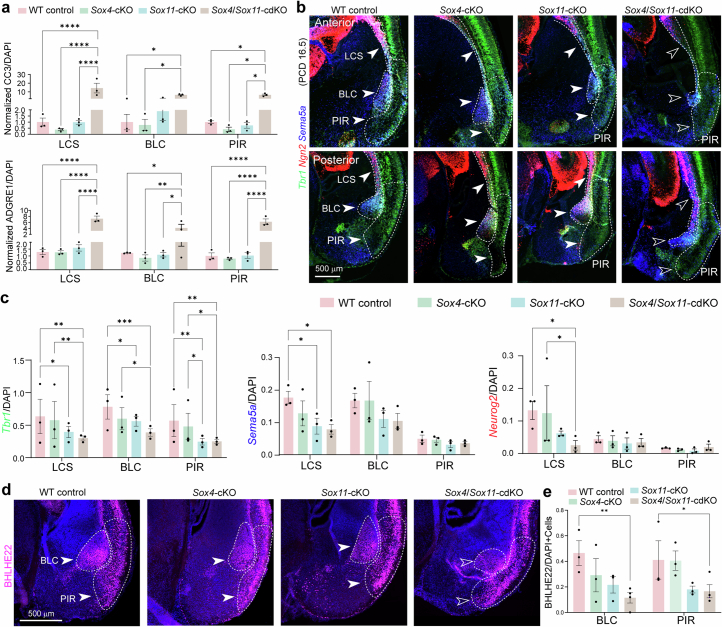
Loss of SOX4 and SOX11 leads to extensive premature apoptosis in the LCS and VLp. **a**) Quantifications of the cleaved caspase3 (CC3)- and ADGRE1-immunopositive cells compared to the DAPI-positive cells in the LCS, BLC and PIR of the WT controls, *Sox4*-cKO, *Sox11*-cKO and *Sox4*; *Sox11*-cdKO. The data is normalized to the WT controls. Two-way ANOVA with multiple comparisons and Tukey’s correction was applied. Mean ± s.e.m. depicted. ****, **, * represents P < 0.0001, 0.01, 0.05 respectively. n = 3/genotype. **b)** Coronal sections from the ventrolateral cortical region of WT controls, *Sox4*-cKO, *Sox11*-cKO and *Sox4*; *Sox11*-cdKO mice brains at PCD 16.5. The LCS, BLC and PIR are depicted using RNAscope for *Tbr1*, *Ngn2*, and *Sema5a*. Filled arrowheads point to normal phenotypes and open arrowheads point to defective phenotypes in the knockouts. **c)** Quantifications of the expression of *Tbr1*, *Ngn2* and *Sema5a* over DAPI in the LCS, BLC and PIR. Two-way ANOVA with multiple comparisons and Tukey’s correction was applied. Mean ± s.e.m. depicted. ***, **,* represents P value < 0.001, 0.01, 0.05, respectively. n = 3/genotype. **d)** Coronal sections from the ventrolateral cortical region of WT controls, *Sox4*-cKO, *Sox11*-cKO and *Sox4*; *Sox11*-cdKO mice brains at PCD 16.5 stained for BHLHE22. Filled arrowheads point to normal phenotype and open arrowhead point to defective phenotypes in the knockouts. **e)** Quantifications of the expression of BHLHE22 over DAPI in the BLC and PIR. Two-way ANOVA with multiple comparisons and Tukey’s correction was applied. Mean ± s.e.m. depicted. **, * represents P value < 0.01 and 0.05, respectively. n = 3/genotype. For detailed statistics, please see Supplementary Table 12.

To determine the cause of PD0 VLp defects, we analysed *Sox4/Sox11*-cdKO mice and littermate controls at PCD16.5. Co-deletion of *Sox4* and *Sox11* induced premature apoptosis along LCS and VLp, as illustrated by increased numbers of cells containing cleaved caspase-3 (CC3), a marker of dying cells, and ADGRE1 (also known as F4-80), which labels infiltration and activation of microglia, compared with littermate wild-type controls, *Sox4*-cKO or *Sox11*-cKO mice (Extended Data Fig. 3a). To investigate whether altered neuronal migration contributed to these defects, we performed RNAscope with probes targeting *Tbr1*, *Neurog2* and *Sema5a* mRNAs, which are markers that have been shown to label LCS, BLC and PIR neurons^11^. In *Sox4/Sox11*-cdKO mice, we observed a significant reduction, but not misrouting, of neurons migrating along the LCS, BLC and PIR primordia that were labelled for *Tbr1*, *Neurog2* and *Sema5a* as well as BHLHE22 (Extended Data Fig. 3b–e). Together, these data suggest that the disruption in the VLp in *Sox4/Sox11*-cdKO mice is driven by prenatal apoptosis rather than neuronal misrouting.

## Conserved expression of *Tfap2d* in VLp excitatory neurons

**Extended Data Fig. 4:**
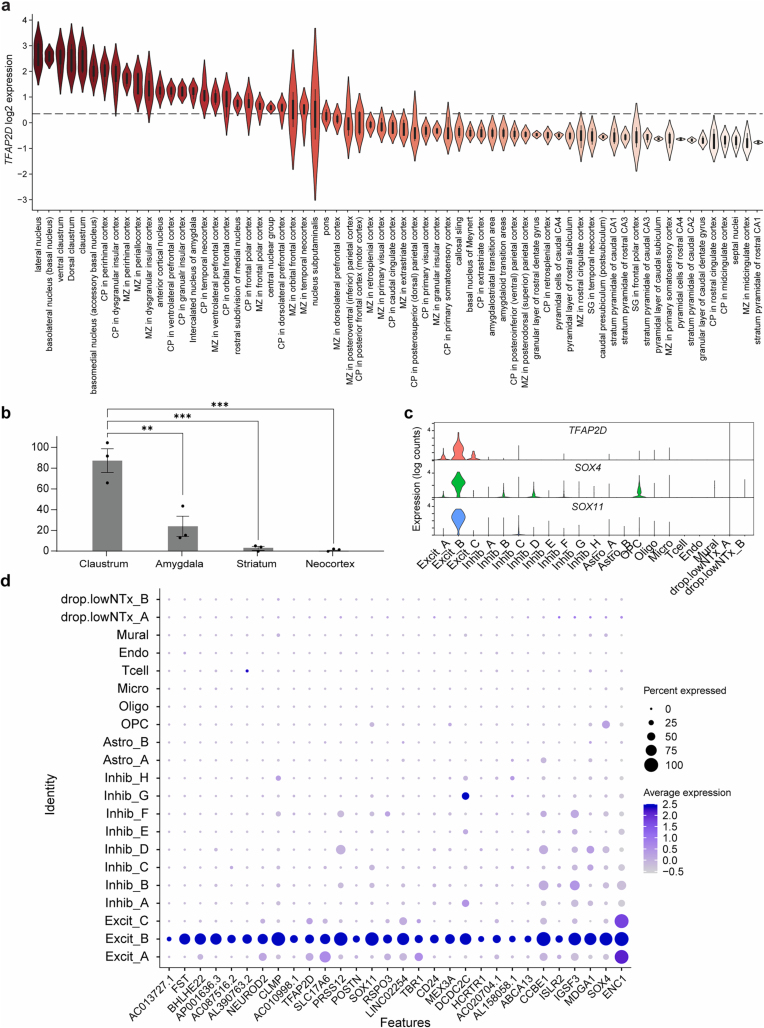
Expression of *TFAP2D* in the human brain. **a**) *TFAP2D* expression pattern across frontal cortical regions, ventrolateral cortical structures, and subcortical regions in prenatal human brain^35^. The dotted line represents the mean of *TFAP2D* expression across all the regions represented here. X-axis depicts the regions and y-axis depicts the log 2 value for the expression pattern. **b**) Barplot showing the expression of *TFAP2D* in the claustrum, amygdala, striatum and neocortex detected by dd-PCR in adult human tissue (n = 3). **c**) Violin plot generated using publicly snRNA-seq available data^36^ showing *TFAP2D*, *SOX4*, and *SOX11* expression in the ExNs (Excitatory neurons) of the human amygdala with highest enrichment in the Excit_B class. y-axis depict the log_2_ expression values and x-axis depicts the different classes of neurons within the human amygdala. **d**) Dot plot showing the percentage of cells that express the top 30 genes within the Excit_B class. Color represents the average expression level and size of the circle represents the percentage of cells. For detailed statistics, please see Supplementary Table 13.

Our previous transcriptomic analyses^23,24,28,29^ revealed an enrichment of *TFAP2D* in the human and non-human primate prenatal amygdala that declines steadily until infancy and persists at lower levels thereafter (Fig. 2a). We corroborated *TFAP2D* expression in the human brain at post-conception week (PCW) 17, specifically in the LCS, as well as in the claustro-amygdalar primordia (Fig. 2b). Furthermore, our analysis of microarray datasets from midfetal and adult human brains^31,32^ revealed a gradient of *TFAP2D* expression, with higher levels observed in BLA, LA, CLA, BMA and PIR, followed by the peripalaeocortical subdivisions of the frontal, insular and temporal cortex, and the orbito-lateral transitional neocortical PFC areas (Fig. 2c,d and Extended Data Fig. 4a). We further confirmed the *TFAP2D* expression pattern in the amygdala and CLA by digital droplet PCR (Extended Data Fig. 4b). Using publicly available human amygdala single-nucleus RNA-seq (snRNA-seq) data^33^, we demonstrated that *TFAP2D* is highly expressed in the amygdala excitatory neurons subclass that also co-expresses *SOX4*, *SOX11*, *TBR1*, *NEUROD2* and *SLC17A6* (Extended Data Fig. 4c,d).

**Extended Data Fig. 5:**
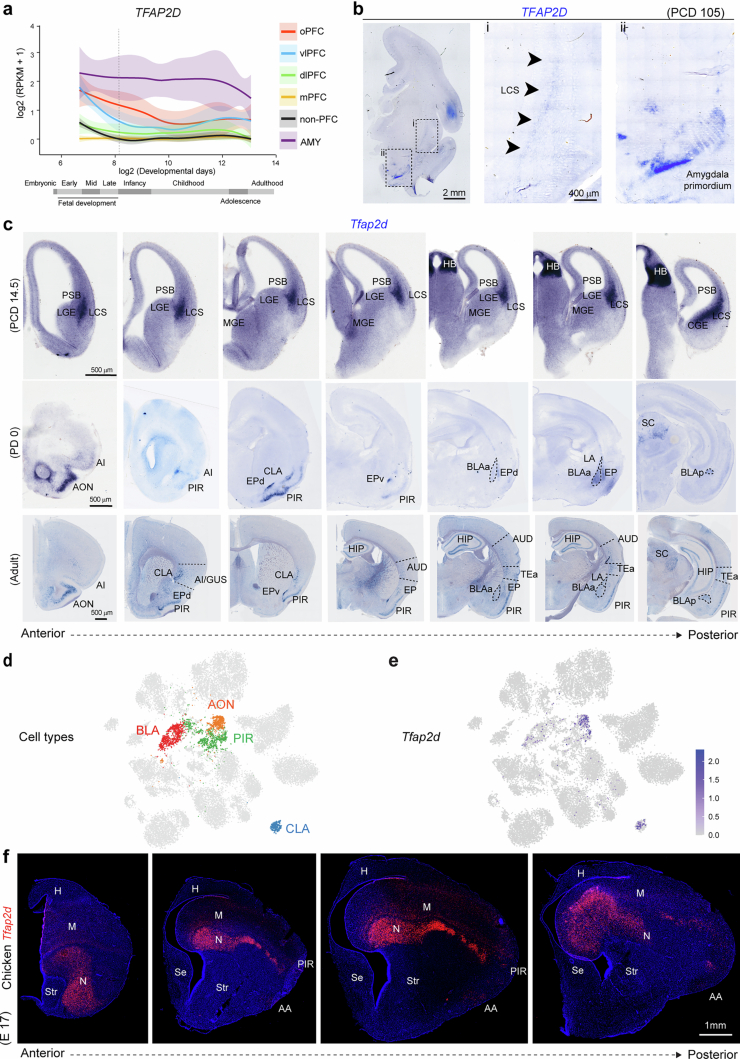
Expression of *Tfap2d* in the macaque and mouse brain. **a**) Plot of *TFAP2D* expression across developmental ages in the macaque AMY, OFC, DFC, VFC, MFC and nonPFC areas within the forebrain^26,27^. Mean ± s.e.m depicted. **b**) Coronal section of a macaque brain at PCD 105 showing *TFAP2D* expression in the LCS, and amygdala primordium detected by in situ hybridization. **c**) Representative serial coronal sections showing *Tfap2d* expression within wildtype mouse PCD 14.5, PD 0 and adult brains detected by in situ hybridization. At PCD 14.5, *Tfap2d* expression is restricted to the LCS and Hb primordium. At PD 0 and in adults *Tfap2d* expression is seen in the BLC, CLA, EPd, EPv PIR, and SC. In adults, deep layer neurons in the AUD, GUS, and TEA show sporadic expression of *Tfap2d*. Representative images from this panel are used in Fig. 2e. **d**-**e**) UMAP showing the subclasses of glutamatergic neurons in the adult mouse cerebrum (left) and the expression of *Tfap2d* within the subclasses representing the BLC, PIR, AON, CLA^37^. **f)** Coronal sections of the E17 chicken brain showing *Tfap2d* (red) expression in the nidopallium and arcopallial amygdala detected by RNAscope. AA, arcopallium amygdala; AON, anterior olfactory nucleus; AUD, auditory cortex; EPv, EP, ventral part; GUS, gustatory cortex; H, hyperpallium; LGE, lateral ganglionic eminence; M, mesopallium; MGE, medial ganglionic eminence; N, nidopallium; SC, superior colliculus; Se, septum; Str, striatum.

RNA-seq datasets sourced from developing and adult macaque and chimpanzee brain regions^23,24^ showed pronounced prenatal expression of *TFAP2D* in the amygdala, which progressively diminishes with advancing age (Extended Data Fig. 5a), similar to the human data. To ascertain the conservation of *TFAP2D* expression patterns in the developing VLp across species, we conducted in situ hybridization experiments in rhesus macaques and mice. We observed *TFAP2D* expression in the macaque LCS and in the amygdala primordium at PCD105 (Extended Data Fig. 5b). In mouse, we performed in situ hybridization at PCD14.5, PD0 and PD60. At PCD14.5, we observed *Tfap2d* in neurons migrating along the LCS and in the amygdala primordium (Fig. 2e and Extended Data Fig. 5c). Notably, *Tfap2d* expression was not observed in the proliferative zones at the PSB (Fig. 2e and Extended Data Fig. 5b,c). This finding indicates that *Tfap2d* expression initiates in immature post-mitotic excitatory neurons migrating along the LCS. At both PD0 and PD60, *Tfap2d* is expressed in the BLC, CLA and anterior insula (Fig. 2e and Extended Data Fig. 5c), resembling humans (Fig. 2c), as well as in the EP and PIR (Fig. 2e and Extended Data Fig. 5c). At PD60, we also observed a sporadic expression pattern of *Tfap2d* in the deep layers of the anterior insula, insular gustatory (GUS), temporal association (TEa) and auditory (AUD) cortex (Extended Data Fig. 5c). We further corroborated this expression pattern in mouse using a publicly available cerebrum snRNA-seq dataset^34^ (Extended Data Fig. 5d,e). Using RNAscope, we detected *Tfap2d* expression in the nidopallium and mesopallium of chicken forebrain on embryonic day (E) 17, including in the PIR and arcopallial amygdala (Extended Data Fig. 5f). These regions are homologues of the mammalian VLp structures, including BLC, CLA and PIR. This evidence indicates that *Tfap2d* expression is highly conserved across a broad spectrum of species, including humans, non-human primates, mice and chickens.

**Extended Data Fig. 6:**
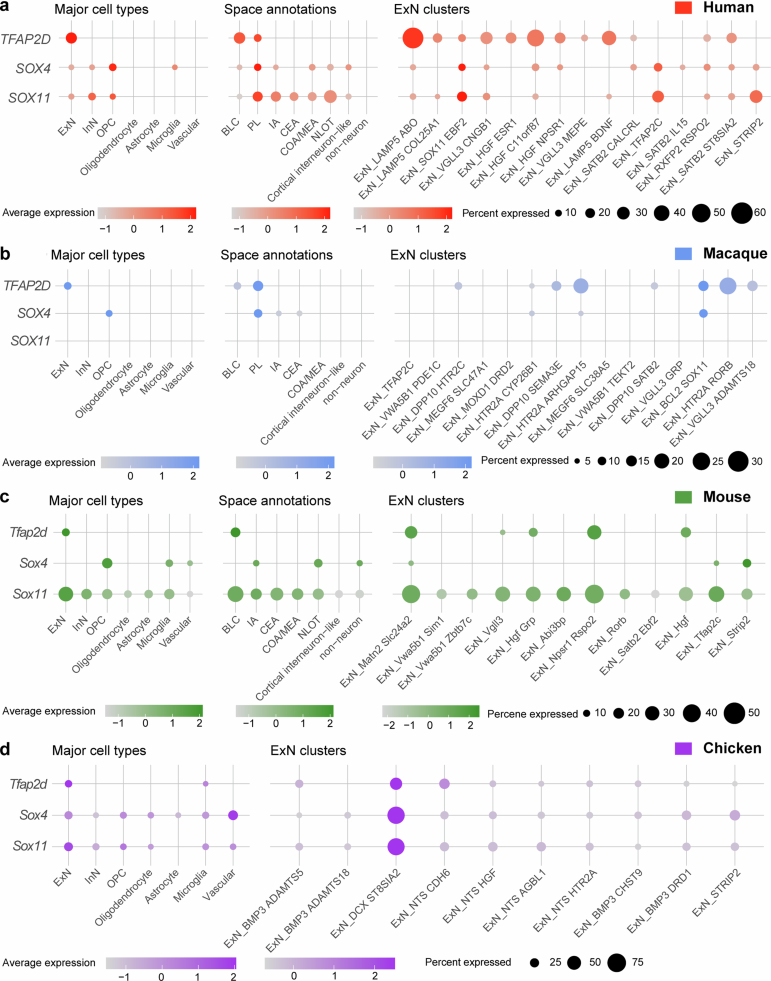
Conserved *Tfap2d* expression in BLC excitatory neurons across mammalian and non-mammalian Species. **a**-**d**) Dot plot annotations showing the expression of *Sox11*, *Sox4* and *Tfap2d* within 1) the major amygdala cell types in all species, 2) the amygdala nuclei in human, macaque, mouse (space annotations), and 3) ExN clusters within the BLC in human, macaque, mouse and within the caudal dorso-ventral ridge (DVR) of chicken, region homologous to mammalian amygdala^38^. In all species, *Tfap2d* exhibited high expression in ExN within the amygdala. IA, intercalated cell masses; InN, Inhibitory neurons; MEA, medial nucleus of the amygdala; OPC, oligodendrocyte progenitor cells; PL, paralaminar nucleus of the amygdala.

**Extended Data Fig. 7:**
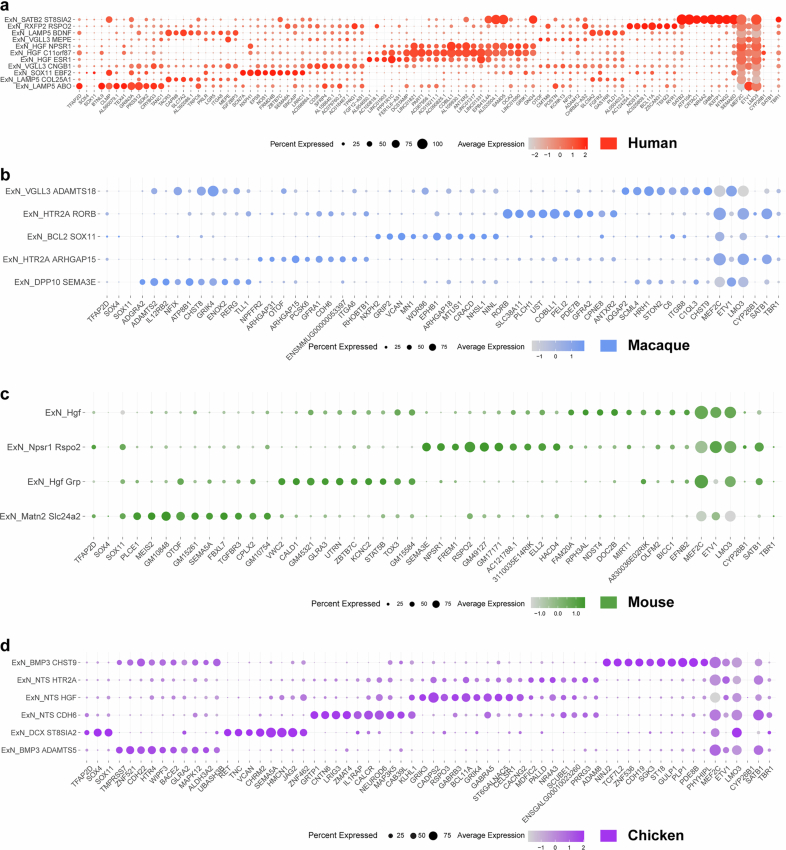
Profiling of *Sox4*, *Sox11* and *Tfap2d* expression in BLC excitatory neurons subclusters across mammalian and non-mammalian species. **a**-**d**) Dot plot annotations showing the top 10 genes marker genes expressed by the ExN clusters *Sox11*, *Sox4* and *Tfap2d* within human, macaque, mouse and chicken^38^.

**Extended Data Fig. 8:**
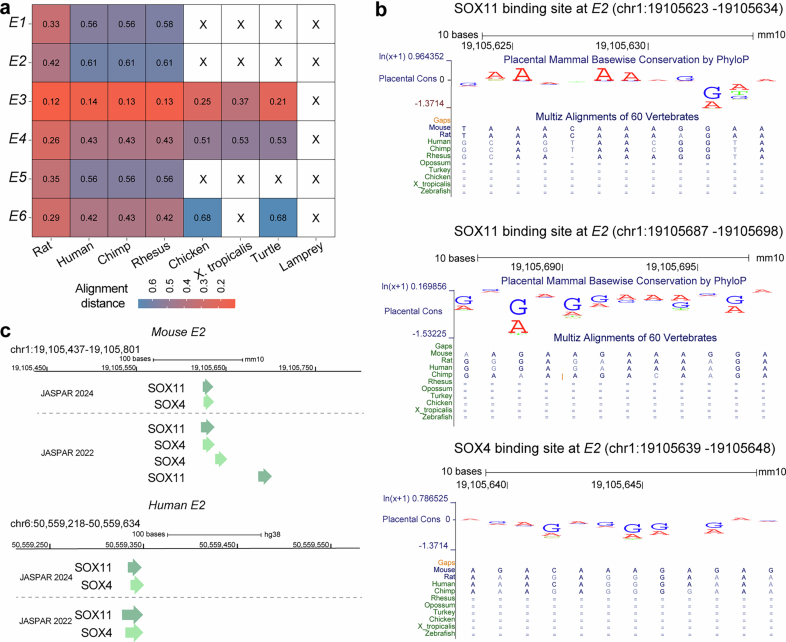
Conservation analysis of *Tfap2d*-associated enhancers across species. **a**) Heatmap illustrating the pairwise alignment distances between various species of vertebrates for the six putative enhancers within the *Tfap2d* locus. X indicates the absence of orthologous sequence. **b**) Line graph for three *SOX11* binding sites within the *Tfap2d-E2* enhancer depicting their conservation across species using MULTIZ alignment. **c**) Line graph displaying the mouse *E2* enhancer and the putative human E2 enhancer, obtained through lift-over of the human genome (hg38). Predictions for *SOX4* and *SOX11* binding sites are included, based on JASPAR 2022 and 2024 release.

Further, our analysis of publicly available amygdala snRNA-seq datasets from human, macaque, mouse and chicken^35^ reveal increased and consistent expression of *Tfap2d* in the BLC and major excitatory neuron subclasses across all profiled species (Extended Data Figs. 6a–d and 7a–d). In human, *TFAP2D* is most highly expressed in *LAMP5* -expressing excitatory neuron clusters (Extended Data Figs. 6a and 7a), which were dominant in the human BLC^35^. Additionally, we observed *SOX4*, *SOX11* and *TFAP2D* expression in the paralaminar nucleus in humans (Extended Data Fig. 6a), which, as previously shown, harbours immature SOX11-positive excitatory neurons in adulthood^17^. In mice, the *Rspo* excitatory neuron subclass exhibited the highest expression of *Tfap2d* and *Sox11* (Extended Data Figs. 6c and 7c), a cell type that is known to regulate anxiety-like behaviours in mice^36^. The similarity of the *TFAP2D* expression pattern across human, macaque, mouse and chicken suggests that there may be a conserved TFAP2D-dependent regulatory network for VLp excitatory neurons specification. In contrast to the conserved co-expression of *Sox4*, *Sox11* and *Tfap2d* expression across species, the SOX4 and SOX11-regulated E2 enhancer, which showed activity in mice, is found exclusively in placental mammals (Extended Data Fig. 8a). The SOX4 and SOX11 binding sites in E2 are partially conserved in placental mammals (Extended Data Fig. 8b). Furthermore, the orthologous human E2 enhancer lacks the putative SOX4 and SOX11 binding sites from mice, but exhibits additional ones as predicted by JASPAR (Extended Data Fig. 8c). Yet, the conserved co-expression of *Tfap2d* with *Sox11* or *Sox4* in BLC excitatory neurons across species hints at potential evolutionary divergence while maintaining a parallel mechanism for *Tfap2d* regulation by SOX4 and SOX11 across species. Together, these results, which corroborate previous findings^37^, suggest that despite changes in SOX4 and SOX11 binding sites across species, *Tfap2d* regulation by SOX4 and SOX11 may be maintained through regulatory mechanisms that are independent of strict DNA sequence constraints.

## *Tfap2d* regulates BLA and LA development

**Fig. 3:**
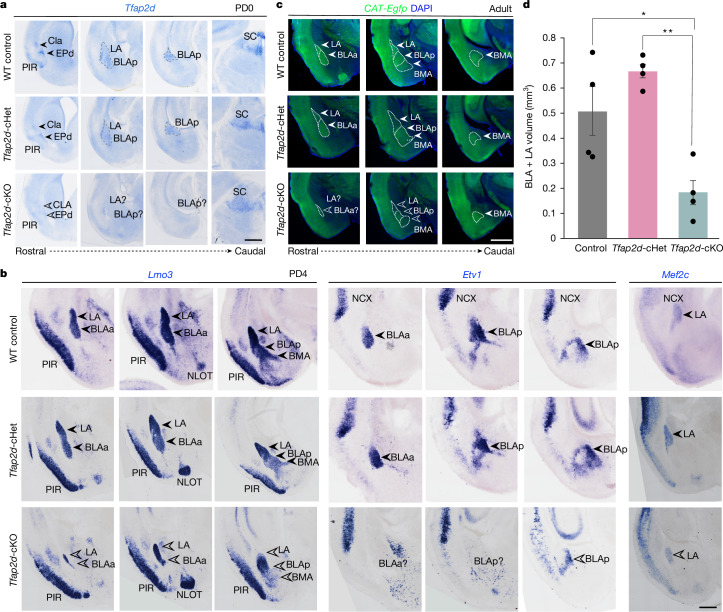
Post-mitotic deletion of *Tfap2d* in cerebral excitatory neurons leads to reduced BLC. **a**, Coronal sections of wild-type control *Tfap2d*-cHet and *Tfap2d*-cKO mice, demonstrating that the specificity of deletion of *Tfap2d* expression is reduced in the VLp (encompassing CLA, EPd, PIR and BLC) but not in the SC in the midbrain. Scale bar, 500 μm. BLAp, BLA, posterior part; SC, superior colliculus. **b**, In situ hybridization for *Lmo3*, *Etv1* and *Mef2c* in coronal sections of wild-type control, *Tfap2d*-cHet and *Tfap2d*-cKO VLp at PD4. Scale bar, 500 μm. NCX, neocortex; NLOT, nucleus of lateral olfactory tract. **c**, Coronal sections of adult wild-type control, *Tfap2d*-cHet and *Tfap2d*-cKO VLp showing the cytoarchitecture of the brain in adults. Scale bar, 1 mm. BLAa, BLA, anterior part. In **a**–**c**, white and black filled arrowheads highlight normal *Tfap2d* expression and open arrowheads highlight loss of *Tfap2d* expression. **d**, Stereological measurements of the combined volume of BLA and LA in adult wild-type control, *Tfap2d*-cHet and *Tfap2d*-cKO brains. Data are mean ± s.e.m. Two-way ANOVA with Tukey’s correction. **P = 0.0022, *P = 0.024; *n* = 4 per genotype. Detailed statistics are presented in Supplementary Table 7.

**Extended Data Fig. 9:**
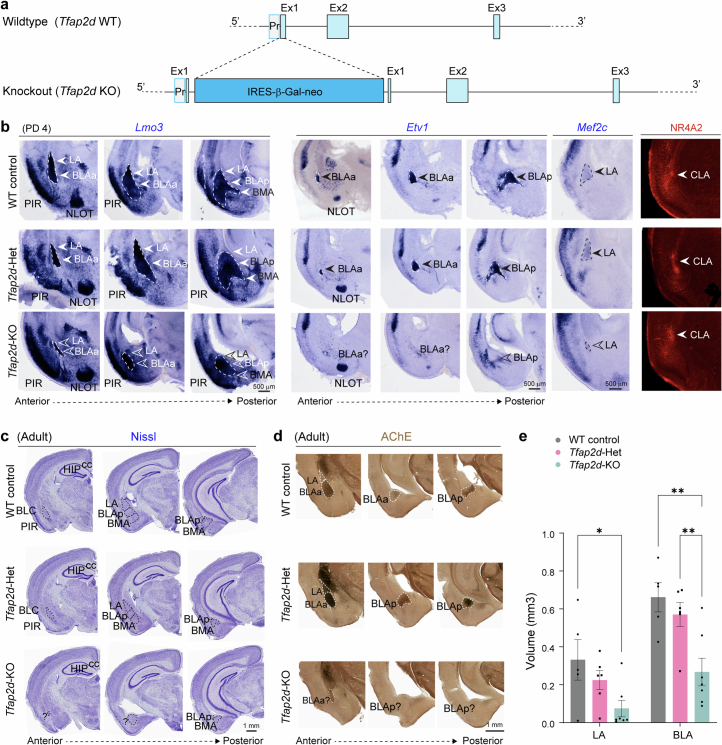
Whole-body knockout of *Tfap2d* results in BLC deficits. **a**) Schematic depicting the *Tfap2d* WT allele and the KO allele with the insertion of LacZ cassette within the exon (Ex) 1^30^. **b**) Coronal sections of the ventrolateral pallial regions of the WT control, *Tfap2d*-Het and *Tfap2d*-KO brains showing the expression of *Lmo3*, *Etv1* and *Mef2c* by in situ hybridizations and NR4A2 by immunostaining at PD 4. Reduction in the size of BLC is seen by *Lmo3*, the most affected region is the *Etv1* labelled BLA. **c**) Nissl staining of the coronal sections of the adult WT control, *Tfap2d*-Het and *Tfap2d*-KO brains. **d**) Coronal sections of the ventrolateral cortical regions of the adult WT control, *Tfap2d*-Het and *Tfap2d*-KO brains showing the AChE staining used to label the BLA. **e**) Stereological measurements of the volume of LA and BLA in the adult WT control, *Tfap2d*-Het and *Tfap2d*-KO brains. Mean ± s.e.m. depicted. Two-way ANOVA with Tukey’s correction was applied. ** and * represents p values < 0.01, <0.05 respectively. n = 5 (WT), 6 (Het), 7 (KO). For detailed statistics, please see Supplementary Table 14.

**Extended Data Fig. 10:**
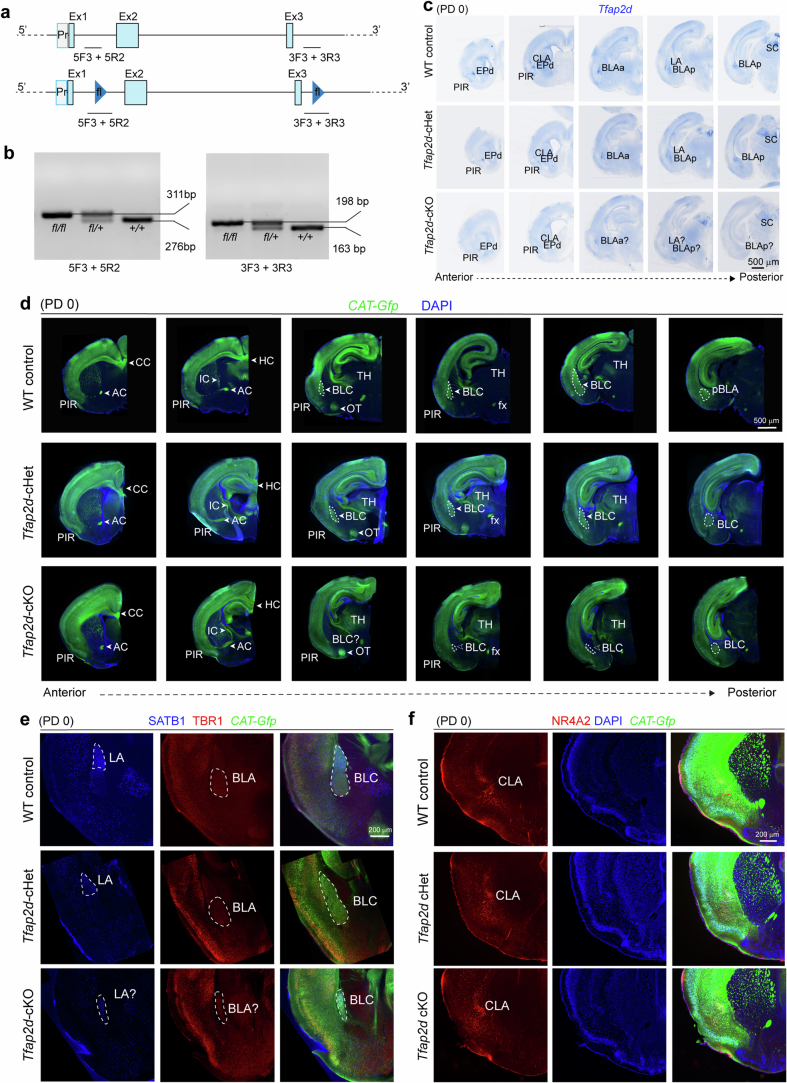
Development of floxed *Tfap2d* allele for conditional post-mitotic deletion of *Tfap2d* in cerebral excitatory neurons that leads to BLC formation deficits. **a**) Schematic depicting the *Tfap2d* wildtype allele (above) and the floxed allele (below) carrying the insertion of flox (fl) cassettes within intron (In) 1 and In 3 of the *Tfap2d* locus. The regions amplified by PCR using genotyping primers are indicated. **b**) Genotyping agarose gel showing the sizes of the amplified PCR products that can distinguish the fl/fl, fl/+ and +/+ genotypes. **c**) Serial coronal sections of WT control, *Tfap2d*-cHet and *Tfap2d*-cKO brains at PD 0 showing reduced expression of *Tfap2d* in the ventrolateral pallial structures (CLA, EPd, PIR, BLC) but not in the midbrain (SC, superior colliculus) of cKO mice. Representative images from this panel are also used in Fig. 3a. **d**) Serial sections of the WT control, *Tfap2d*-cHet and *Tfap2d*-cKO brains at PD 0 depicting their gross morphology via visualization of GFP expression driven by the *Neurod6* (*Nex1*)-Cre driver. **e**) Immunostaining showing the expression of TBR1 and SATB1 in the WT control, *Tfap2d*-cHet and *Tfap2d*-cKO brains at PD 0, highlighting the deficits seen in the BLC. **f**) Immunostaining showing the expression of NR4A2 in the CLA of WT control, *Tfap2d*-cHet and *Tfap2d*-cKO brains at PD 0. Filled arrows indicate normal morphology whereas open arrows indicated altered BLC structure.

To examine the effect of *Tfap2d* deletion on VLp development, we analysed mice with a targeted disruption of *Tfap2d*. We first investigated *Tfap2d* lacZ mice, in which a β-galactosidase–neomycin cassette, interrupting exon 1 of the *Tfap2d* gene, abolishes *Tfap2d* expression^30^ (Extended Data Fig. 9a). Additionally, given the previously reported *Tfap2d* expression and function in the superior colliculus^30^, we also developed *Tfap2d*-floxed mice (Extended Data Fig. 10a,b and Supplementary Table 3). We crossed these mice with *Neurod6-cre*;CAT-Gfp mice to delete *Tfap2d* in post-mitotic cerebral excitatory neurons, yielding conditional mutant groups (*Tfap2d*-cKO, *Tfap2d* conditional heterozygous (cHet) and wild-type control) that express a GFP reporter in cerebral excitatory neurons. In *Tfap2d*-knockout (KO) and *Tfap2d*-cKO mice, *Tfap2d* expression was absent in VLp, including in the BLC, PIR, CLA and EP, however, expression in the superior colliculus remained unaffected in the *Tfap2d*-cKO mice (Fig. 3a and Extended Data Fig. 10c). Mice of all these genotypes survived to adulthood.

To assess whether VLp development is altered following *Tfap2d* loss, we conducted a comprehensive analysis of perinatal mouse brains. No notable defects were observed in neocortical or archicortical structures, including in GFP-labelled long-range subcerebral and interhemispheric projections in the *Tfap2d*-cKO mice (Extended Data Fig. 10d), aligning with the restricted expression pattern of *Tfap2d* to claustro-amygdalar and palaeocortical excitatory neurons. However, Nissl and acetylcholinesterase staining in *Tfap2d*-KO and GFP expression in *Tfap2d*-cKO indicated a marked reduction in BLC size (Fig. 3c,d and Extended Data Fig. 9c–e). Further, immunostaining for TBR1 and SATB1 (Extended Data Fig. 10e) and in situ hybridization for *Lmo3*, *Etv1* and *Mef2c* (Fig. 3b and Extended Data Fig. 9b), confirmed a near-complete loss of BLA, partial loss of LA, but no obvious loss of BMA in *Tfap2d*-KO and *Tfap2d*-cKO mice compared with their respective heterozygous and wild-type controls. Immunostaining for NR4A2, which labels CLA excitatory neurons, showed no noticeable differences in CLA across genotypes (Extended Data Figs. 9b and 10f). In adults, no appreciable anatomical abnormalities were observed in other VLp structures in *Tfap2d*-cKO or *Tfap2d*-KO mice (Fig. 3c and Extended Data Fig. 10c,d). These findings indicate that *Tfap2d* has an essential cell-autonomous role in the post-mitotic development of LA and BLA excitatory neurons.

**Extended Data Fig. 11:**
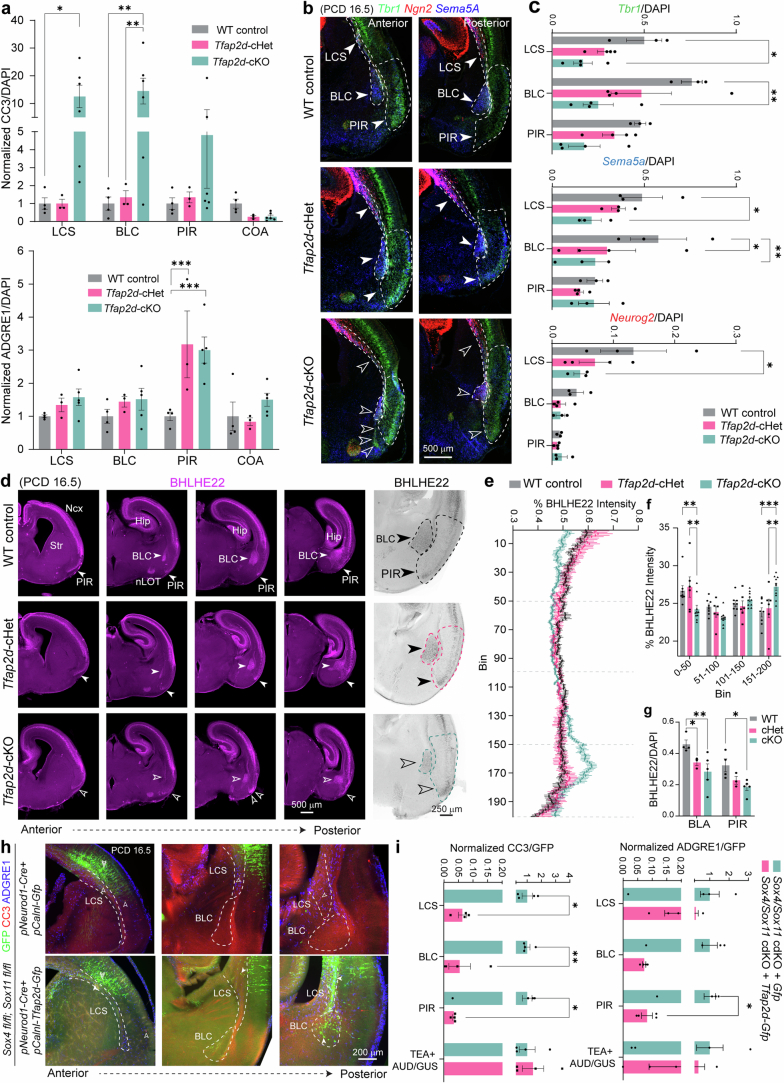
Loss of *Tfap2d* leads to premature apoptosis in the LCS and VLp downstream of *Sox4* and *Sox11.* **a**) Quantifications of the CC3-positive and ADGRE1-positive cells over the DAPI-positive cells detected in the LCS, BLC and PIR of the WT controls, *Tfap2d*-cHET, and *Tfap2d*-cKO. The data is normalized to the WT controls. Two-way ANOVA with multiple comparisons and Tukey’s correction was applied. Mean ± s.e.m. depicted. **, * represents P < 0.01, 0.05. n = 4 (WT), 3(cHET), 6(cKO) for CC3 and 4(WT), 3(cHET) and 5(cKO) for ADGRE1. **b**) Coronal sections from the ventrolateral cortical region of WT controls, *Tfap2d*-cHET, and *Tfap2d*-cKO mice brains at PCD 16.5. The LCS, BLC and PIR are depicted using RNAscope for *Tbr1*, *Ngn2*, and *Sema5a*. Filled arrowheads point to normal phenotype and open arrowhead point to defective phenotypes in the knockouts. **c**) Quantifications of the expression of *Tbr1*, *Ngn2* and *Sema5a* over DAPI in the LCS, BLC and PIR. Two-way ANOVA with multiple comparisons and Tukey’s correction was applied. Mean ± s.e.m. depicted. **, * represents P < 0.01, 0.05. n = 3 (WT), 4(cHET), 4(cKO) for *Tbr1* and 3(WT), 4(cHET) and 3(cKO) for *Sema5a* and *Ngn2*. **d**) Coronal sections from WT controls, *Tfap2d*-cHET, and *Tfap2d*-cKO mice brains at PCD 16.5 stained for BHLHE22. Filled arrowheads point to normal phenotype and open arrowhead point to defective phenotypes in the knockouts. **e**) Quantifications of the BHLHE22 intensity per bin normalized to total intensity with PIR. Two-way ANOVA with multiple comparisons and Tukey’s correction was applied. Mean ± s.e.m. depicted. P_interaction_ < 0.0001 n = 8 (WT), 6(cHET), 10(cKO). **f**) Quantifications of the BHLHE22 intensity per 50 bins normalized to total intensity with PIR. Two-way ANOVA with multiple comparisons and Tukey’s correction was applied. Mean ± s.e.m. depicted. ***, ** represents P value < 0.001, 0.01. n = 8 (WT), 6(cHET), 10(cKO). **g**) Quantifications of the BHLHE22 ExNs over the DAPI-positive cells detected in the BLC and PIR of the WT controls, *Tfap2d*-cHET, and *Tfap2d*-cKO. Two-way ANOVA with multiple comparisons and Tukey’s correction was applied. Mean ± s.e.m. depicted. **, * represents P < 0.01, 0.05. n = 4 (WT), 3 (cHET), 5 (cKO). **h**) Coronal sections from *Sox4* fl/fl; *Sox11* fl/fl mice brains at PCD 16.5 stained for GFP, CC3 and ADGRE1 that were injected with *p Neurod1-Cre* and *pCalnl-Gfp* (negative control) or *pCalnl-Tfap2d-ires-Gfp* (*Tfap2d* rescue) at PCD 12.5 by *in utero* electroporation. Open arrowheads point to CC3 positive dying cells whereas filled arrowheads point to the GFP positive cells migrating along the LCS and reaching BLC. Double empty arrowheads point to CC3 and GFP positive dying cells in the adjacent cortex **i**) Quantifications of the CC3 positive and ADGRE1 positive cells over the GFP positive cells detected in the LCS, BLC, PIR and ventrolateral cortical regions (AUD/GUS + TEA). The data is normalized to the GFP positive cells seen in the negative controls. Multiple unpaired two-tailed t-test was applied. Mean ± s.e.m. depicted. **, * represents P < 0.01, 0.05. n = 3 (negative control), n = 4 (*Tfap2d* rescue). For detailed statistics, please see Supplementary Table 15.

To determine the origin of the developmental deficits in *Tfap2d*-cKO BLA, we conducted a detailed analysis of cell death and migration defects, similar to the analysis performed with *Sox4/Sox11*-cdKO mice. Similar to *Sox4/Sox11*-cdKO mice, *Tfap2d*-cKO brains showed increased cell death and activated microglia compared with littermate wild-type and *Tfap2d*-cHet brains (Extended Data Fig. 11a). This increased cell death resulted in fewer neurons migrating along the LCS and in BLC and PIR, as labelled by BHLHE22, *Tbr1*, *Neurog2* and *Sema5a* (Extended Data Fig. 11b–d,g). However, unlike *Sox4/Sox11*-cdKO mice, *Tfap2d*-cKO mice also exhibited migration defects, specifically in the ventral LCS. A small stream of *Ngn2*-positive excitatory neurons (*Ngn2* typically labels LCS and BLC neurons), which also express *Tbr1*, reached the ventromedial PIR (Extended Data Fig. 11b), unlike in littermate wild-type controls and *Tfap2d*-cHet mice. Furthermore, BHLHE22 staining revealed increased clustering at the ventromedial PIR in *Tfap2d*-cKO mice compared with littermate controls (Extended Data Fig. 11d–g). These findings suggest that *Tfap2d* deletion results in premature apoptosis of some LCS, BLC and PIR excitatory neurons. Additionally, some excitatory neurons exhibited migration defects that rather than entering BLC primordia, continued to migrate ventrally towards the PIR. These early embryonic migration defects may also cause potential alterations in the VLp connections and functioning.

The predisposition of *Sox4/Sox11*-cdKO cells to undergo apoptosis (Extended Data Fig. 3a) may be due to disruption of *Tfap2d* expression. To access this, we selectively deleted *Sox4* and *Sox11* expression while concurrently rescuing *Tfap2d* in a subset of post-mitotic excitatory neurons originating at the PSB, which migrate to the BLC, PIR and neighbouring cortical regions (AUD/GUS and TEA) by performing in utero electroporation at PCD12.5. As negative controls, we performed similar *Sox4* and *Sox11* deletion with no rescue (Extended Data Fig. 11h). Analysis at PCD16.5, showed a markedly reduced number of CC3-positive dying cells in the LCS, BLC and PIR, but not in neighbouring cortical regions, of *Tfap2d*-rescued brains compared with negative controls (Extended Data Fig. 11h,i). GFP-negative cells also underwent apoptosis, probably owing to the loss of GFP expression in dying cells or cell-extrinsic factors. These findings suggest that in LCS, BLC and PIR, *Tfap2d* prevents *Sox4* and *Sox11* deletion-associated apoptosis. However, in the neocortex, *Sox4* and *Sox11* operate through *Tfap2d*-independent pathways.

## *Tfap2d* loss or haploinsufficiency disrupts BLC–PFC connections

**Fig. 4:**
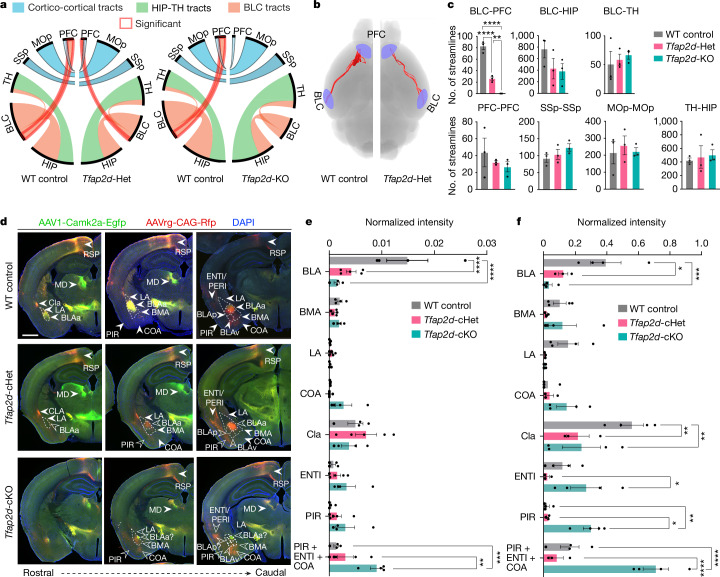
*Tfap2d* loss or haploinsufficiency alters connections between BLC and PFC. **a**, Streamlines generated as a connectivity measurement between the BLC and mPFC, HIP and TH at PD120–PD180 in *Tfap2d*-KO mice using DTI (*n* = 3 per genotype). MOp, primary motor cortex; SSp, primary somatosensory cortex. **b**, Visualization of streams connecting the BLC and mPFC in wild-type control and *Tfap2d-*Het brains. **c**, Number of streamlines between BLC and mPFC, HIP and TH and between contralateral cortical areas in wild-type control, *Tfap2d*-Het and *Tfap2d*-KO mice. One-way ANOVA with Bonferroni’s multiple comparisons test. ****P < 0.0001, **P = 0.0074; *n* = 3 per genotype. **d**, Representative images of wild-type control, *Tfap2d*-cHet and *Tfap2d*-cKO brains with efferent and afferent projections traced from the mPFC using AAV1-Camk2a-Egfp (anterograde) and AAVrg-CAG-Rfp (retrograde) at PD90. Open arrowheads indicate reduced or misdirected projections from and to the mPFC; filled arrowheads indicate typical projection pattern from mPFC. Scale bar, 500 μm. BLAv, BLA, ventral; MD, mediodorsal nucleus of the TH; RSP, retrosplenial cortex. **e**,**f**, Normalized intensity of retrograde tracings (**e**; AAVrg-CAG-Rfp labelling in **d**) and anterograde tracings (**f**; AAV1-Camk2a-Egfp labelling in **d**) in VLp, including BLC, CLA, COA, PIR and ENTI/PERI in wild-type control, *Tfap2d*-cKO and *Tfap2d*-cHet mice. Data are mean ± s.e.m. Two-way ANOVA with Tukey’s correction. **e**, PIR + ENTI + COA, *Tfap2d*-cHet versus *Tfap2d*-cKO: **P = 0.0028. ***P = 0.0002; ****P < 0.0001. *n* = 4 (wild type), 5 (cHet) and 4 (cKO) mice. **f**, BLA, wild type versus *Tfap2d*-cHet: *P = 0.0129; ENTI, *Tfap2d*-cHet versus *Tfap2d*-cKO: *P = 0.025; PIR, *Tfap2d*-cHet versus *Tfap2d*-cKO: *P = 0.0115; Cla, wild type versus *Tfap2d*-cHet: **P = 0.0012; Cla, wild type versus *Tfap2d*-cKO: **P = 0.0011; PIR, wild type versus *Tfap2d*-cHet: **P = 0.0051. ***P = 0.0002; ****P < 0.0001. *n* = 4 (wild type), 3 (cHet) and 4 (cKO). Detailed statistics are presented in Supplementary Table 8.

BLC excitatory neurons form extensive long-range connections to multiple brain regions, including the PFC, thalamus (TH) and hippocampus^38^, which encode salient cues from the environment^1–3,5^. Prompted by the observed reduction of BLC in *Tfap2d*-deficient mice, we investigated the influence of *Tfap2d* expression on long-range axonal projections from BLC. Using diffusion tensor imaging (DTI) on control, *Tfap2d*-heterozygous (Het) and *Tfap2d*-KO brains, we found a marked impairment of BLC–PFC connectivity in *Tfap2d*-KO mice (Fig. 4a). Notably, despite a significant reduction in BLC–PFC connectivity in *Tfap2d*-Het brains compared with wild-type controls, these brains exhibited significantly increased connectivity relative to *Tfap2d*-KO brains, indicating a dosage-dependent, intermediate effect of *Tfap2d* haploinsufficiency on BLC–PFC connections (Fig. 4c). However, we observed no significant differences in BLC–TH, BLC–hippocampus, or contralateral cortical–cortical connections in the somatosensory cortex, primary motor cortex and PFC across the genotypes (Fig. 4a–c). This suggests a targeted effect of *Tfap2d* deficiency on specific BLC-related neural pathways.

**Extended Data Fig. 12:**
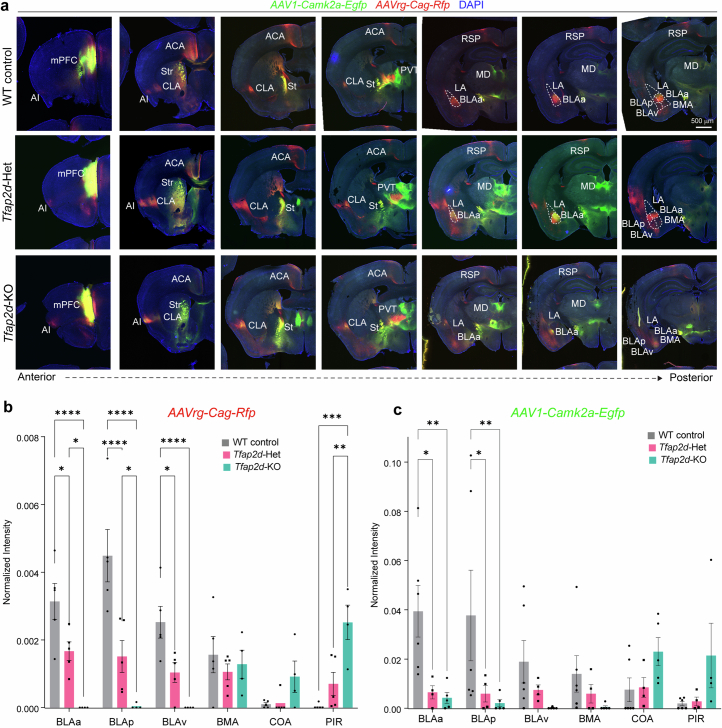
*Tfap2d* loss or haploinsufficiency impairs BLC-mPFC connectivity. **a**) Serial coronal sections of WT control, *Tfap2d*-Het and *Tfap2d*-KO adult brains whose efferent and afferent projections from the mPFC were traced using *AAV1-Camk2a-Egfp* (green; anterograde) and *AAVrg-Cag-Rfp* (red; retrograde), respectively. **b**-**c**) Bar graphs depicting the normalized intensity of the number of cells carrying *AAVrg-Cag-Rfp* tracer (b) or mean intensity fluorescence for the axons labelled with *AAV1-Camk2a-Egfp* (c) normalized to the intensity of the tracer at injection sites in various ventrolateral cerebral brain regions of control, *Tfap2d*-Het and *Tfap2d*-KO adult mice. Mean ± s.e.m. depicted. Two-way ANOVA with Tukey’s correction was applied. ****P < 0.0001,***P = 0.0001, **P = 0.0059 (PIR[b], HET vs KO; = 0.0039 BLAa[c], WT vs KO; =0.0034 BLAp[c], WT vs KO *P = 0.0208, 0.0124 (BLAa [b], WT vs HET, HET vs KO); =0.0309 (BLAp[b], 0.019 (BLAv[b] WT vs HET; = 0.0128 (BLAa [c] WT vs HET; = 0.0169 (BLAp [c] WT vs HET). (n = 5 (WT), 5 (Het), 4 (KO). Str, striatum; St, stria terminalis; PVT, paraventricular thalamus; ACA, anterior cingulate area. For detailed statistics, please see Supplementary Table 16.

To further corroborate the deficits in BLC–PFC connectivity observed by DTI, we used adeno-associated virus (AAV) based tract tracing in *Tfap2d*-deficient mice. Given the significant reduction in BLC size in *Tfap2d*-deficient mice, we introduced both anterograde and retrograde AAV tracers into the medial PFC (mPFC), broadly targeting both the prelimbic (PL) and infralimbic (ILA) areas, regions that showed significant alterations in BLC connectivity in the DTI analysis. Consistent with the DTI observations, all genotypes with reduced or absent *Tfap2d* expression (*Tfap2d*-cHet, *Tfap2d*-HET, *Tfap2d*-cKO and *Tfap2d*-KO) exhibited a significant decrease in BLA–mPFC and mPFC–BLA projections compared with wild-type controls (Fig. 4d–f and Extended Data Fig. 12a–c). In addition to the reduced BLA–mPFC connections, we observed a dispersed projection pattern from the cortical amygdalar area (COA), PIR and perirhinal cortex to the PFC in *Tfap2d*-KO and *Tfap2d*-cKO mice compared with wild-type counterparts (Fig. 4d–f and Extended Data Fig. 12a–c). This shift in projection patterns is probably due to the migration defects in prenatal excitatory neurons causing their aberrant accumulation in ventral PIR (Extended Data Fig. 11b–g) in *Tfap2d*-deficient mice. In addition to a general decrease in BLA–PFC connectivity, *Tfap2d*-Het and *Tfap2d*-cHet mice also exhibited individual variations in atypical projection patterns involving the PIR, COA and PFC. Collectively, these findings underscore that the absence of one or both *Tfap2d* alleles results in altered BLA–mPFC connections, highlighting the critical role of *Tfap2d* gene dosage in maintaining proper neural circuitry.

## *Tfap2d*-related BLC deficits increase threat response

**Extended Data Fig. 13:**
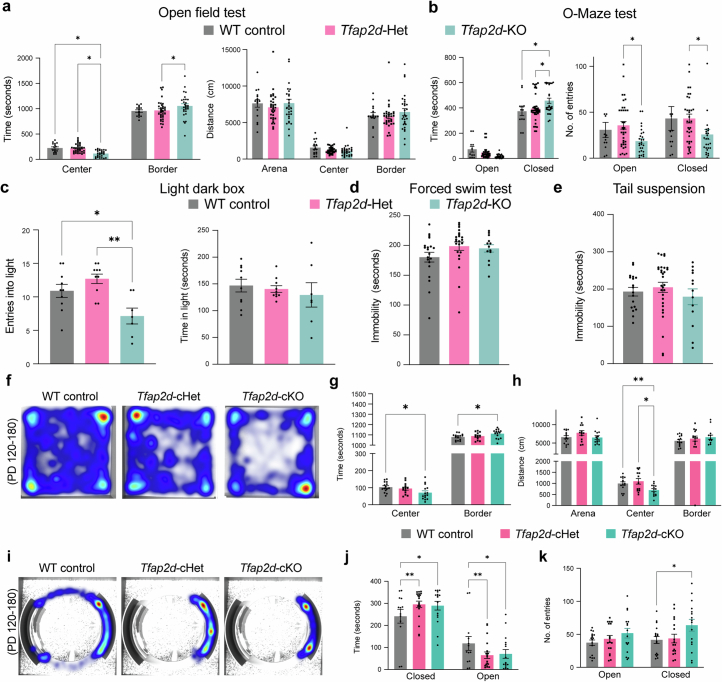
Loss or Haploinsufficiency of *Tfap2d* alters non-Learned exploratory behaviors. **a**) Bar graph showing the cumulative time spent (sec) and distance moved (cm) in the center vs border analysis. *Tfap2d*-KO spent significantly more time in the border and moved less in the center, as compared to the WT control, and *Tfap2d*-Het. Mean ± s.e.m. depicted. Two-way ANOVA with Tukey’s correction was applied. * represents p values, <0.05. n = 16 (WT control), 33 (*Tfap2d*-Het), and 29 (*Tfap2d*-KO). **b**) Cumulative time spent (seconds) and number (#) of entries in the open arm vs closed arm analysis of the o-maze test. *Tfap2d*-KO animals spent significantly more time in the closed arm and had more entries in the closed arm as compared to the WT control and or *Tfap2d*-Het animals. Mean ± s.e.m. depicted. Two-way ANOVA with Tukey’s correction was applied. ** represents p values < 0.05. n = 14 (WT control), 38 (*Tfap2d*-Het), 27 (*Tfap2d*-KO). **c**) Number of entries and cumulative amount of time that spent on the light side of the light-dark box. One-way ANOVA with Tukey’s correction was applied. ** and * represents p values < 0.01, <0.05 respectively. n = 10 (WT control), 10 (*Tfap2d*-Het), and 7 (*Tfap2d*-KO). **d**) Cumulative amount of time that animal spent immobile in the forced swim test. One-way ANOVA with Tukey’s correction was applied. n = 20 (WT control), 23 (*Tfap2d*-Het) and 13 (*Tfap2d*-KO). **e**) Cumulative amount of time that animal spent immobile in the tail suspension test. One-way ANOVA with Tukey’s correction was applied. n = 18 (WT control), 24 (*Tfap2d*-Het), and 13 (*Tfap2d*-KO). For detailed statistics, please see Supplementary Table 17. **f)** Representative heatmaps showing the cumulative movement of WT control, *Tfap2d*-cHet and *Tfap2d*-cKO animals in the open field test for 20 min. **g-h)** Cumulative time spent (b) and distance moved (c) in the center vs border analysis, showing the *Tfap2d*-cKO spent significantly more time in the border and moved less in the center, as compared to the WT control, and *Tfap2d*-cHet animals. Mean ± s.e.m. depicted. Two-way ANOVA with Tukey’s correction was applied. ** and * represent p values < 0.01, <0.05 respectively. n = 14 (WT control), 14 (*Tfap2d*-cHet) and 15 (*Tfap2d*-cKO). **i)** Representative heatmap showing the cumulative movement of WT control, *Tfap2d*-cHet and *Tfap2d*-cKO animals in the o maze test for 6 min. **j-k)** Cumulative time spent and number of entries in the open arm vs closed arm analysis, showing the *Tfap2d*-cKO and *Tfap2d*-cHet spent significantly more time in the closed arm and *Tfap2d*-cKO had more entries in the closed arm as compared to the WT control. Mean ± s.e.m. depicted. Two-way ANOVA with Tukey’s correction was applied. ** and * represents p values < 0.01, <0.05 respectively. n = 19 (WT control), 13 (*Tfap2d*-cHet) and 16 (*Tfap2d*-cKO). For detailed statistics, please see Supplementary Table 18.

The BLC has a crucial role in encoding the valence of environmental cues, transmitting this critical information to other brain regions tasked with triggering behavioural responses^5^. To investigate the effect of *Tfap2d*-driven BLC-related deficits on adult exploratory behaviour, we conducted open-field and zero-maze behavioural tests on adult *Tfap2d*-KO and *Tfap2d*-cKO mice and their wild-type and *Tfap2d*-Het littermates (Extended Data Fig. 13a,b,f–k). The findings reveal that *Tfap2d*-KO and *Tfap2d*-cKO mice exhibit a tendency to spend less time in the centre of the open field relative to their wild-type and *Tfap2d*-Het littermates (Extended Data Fig. 13a,f–h). All genotypes travelled the same distance, indicating no gross motor deficits in the *Tfap2d*-KO or *Tfap2d*-cKO mice (Extended Data Fig. 13a,h). In the zero-maze test, *Tfap2d*-KO, *Tfap2d*-cKO and *Tfap2d*-cHet mice spent more time in the closed arm of the zero maze compared with their respective wild-type groups (Extended Data Fig. 13b,i–k). *Tfap2d-*KO mice exhibited fewer entries to the light side of the light-dark box test than wild-type or *Tfap2d*-Het mice but displayed no differences in the forced swim test and tail suspension test, behavioural assays related to depression-like behaviours (Extended Data Fig. 13c–e). These data demonstrate that *Tfap2d*-dependent deficits in BLC formation and connectivity leads to alterations in threat behaviours in adults, in which *Tfap2d*-KO mice consistently avoided stressful areas across various behavioural tests. This indicates that the lack of *Tfap2d* during development promotes heightened ‘trait’ anxiety-like behaviour. The absence of differences in the forced swim test and tail suspension test suggests that this is specific to anxiety, and does not include depression-like behaviours. Anxiety-like behaviour is often linked to amygdalar hyperexcitability^39^, which aligns with disrupted BLA–PFC connections in *Tfap2d*-cKO mice, suggesting that reduced PFC activity may leads to increasing amygdala activity, stress reactivity and anxiety^5,6^.

**Fig. 5:**
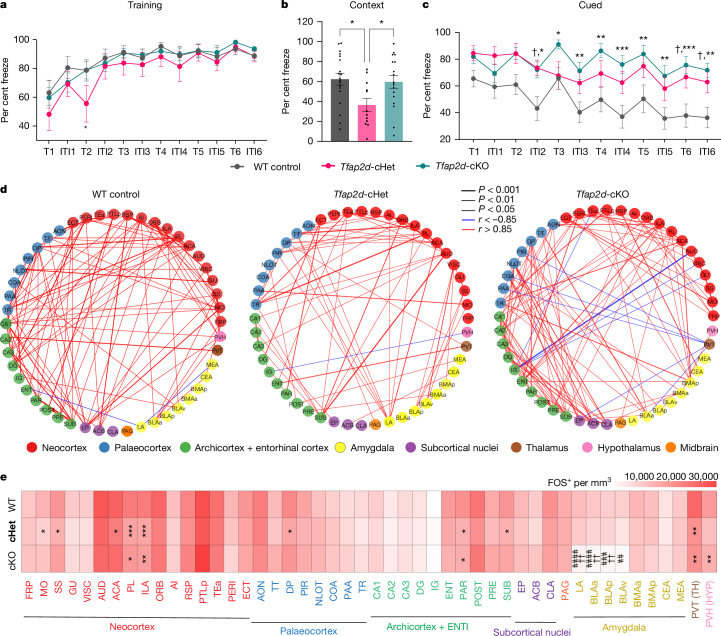
*Tfap2d* loss or haploinsufficiency increases threat responding in learned behaviours and alters functional connectivity. **a**, The percentage of time wild-type control, *Tfap2d*-cHet and *Tfap2d*-cKO mice freeze during the learning (training) period of the fear conditioning test during inter-tone interval (ITI) and tone (75 decibels, 20 s) (T) followed by a 2-s shock at 0.5 mA. **b**, The percentage of time wild-type control, *Tfap2d*-cHet and *Tfap2d*-cKO mice freeze during conditioned responses while in the training context. One-way ANOVA with Tukey’s correction. *P < 0.05. **c**, The percentage of time wild-type control, *Tfap2d*-cHet and *Tfap2d*-cKO mice freeze during trial and ITI without shock during cued responses. Data are mean ± s.e.m. Two-way ANOVA with repeated measures and multiple comparison Tukey’s correction. ***P < 0.001, **P < 0.01, *P < 0.05 for wild type versus *Tfap2d*-cKO; ^†^*P* < 0.05 for wild type versus *Tfap2d*-cHet. *n* = 16 (wild-type control), 12 (*Tfap2d*-cHet) and 16 (*Tfap2d*-cKO). Detailed statistics are presented in Supplementary Table 9. **d**, Network graph depicting interregional correlation between brain regions as measured by the number of FOS-positive cells in wild-type control, *Tfap2d*-cHet and *Tfap2d*-cKO brains. Nodes, represent brain regions, edges indicate significant correlation scores (*P* < 0.05 and *r* > 0.85) and colours correspond to broader brain areas. Detailed statistics are presented in Supplementary Table 10. **e**, Heat map depicting the number of FOS-positive cells detected per unit area in the different brain regions of the wild-type control, *Tfap2d*-cHet and *Tfap2d*-cKO brains isolated after 60–90 min of cued test. Data are mean ± s.e.m. Two-way ANOVA with Tukey’s correction. ***P < 0.001, **P < 0.01, *P < 0.05 for *n* = 4 per genotype. LA, BLAa, BLAp and BLAv, wild type versus *Tfap2d*-cKO: ^####^*P* < 0.0001, ^###^*P* < 0.001, ^##^*P* < 0.01. *Tfap2d*-cHet versus *Tfap2d*-cKO: ^††^*P* < 0.01. Detailed statistics are presented in Supplementary Table 11. Brain region abbreviations for **d**,**e** are listed in Supplementary Table 4.

**Extended Data Fig. 14:**
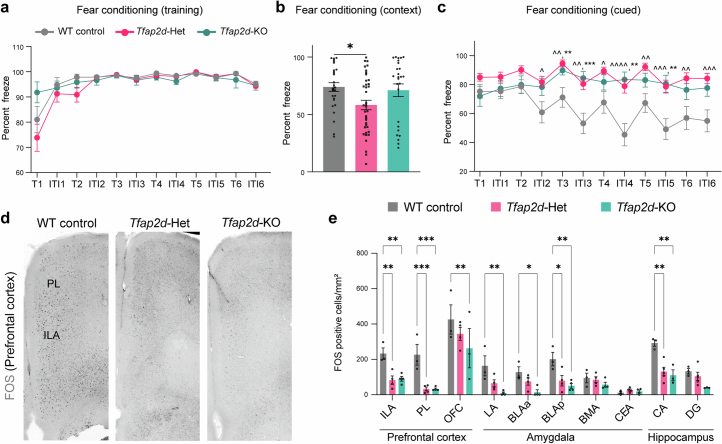
Whole-body *Tfap2d* loss or haploinsufficiency alters threat response in the fear conditioning test. **a**) Line plot showing that during learning phase WT control, *Tfap2d* Het and *Tfap2d* KO mice freeze for same amount of time by tone 6. Mean ± s.e.m. depicted. Two-way ANOVA with Tukey’s correction was applied. **b**) Bar plot showing conditioned responses of the WT control, *Tfap2d*-cHet and *Tfap2d*-cKO mice as percent of time the animals froze while in the training context. Mean ± s.e.m. depicted. One-way ANOVA with Tukey’s correction for multiple comparisons was applied. * represent p values < 0.05. **c**) Line plot showing cued responses of the WT control, *Tfap2d*-cHet and *Tfap2d*-cKO mice. Mean ± s.e.m. depicted. Two-way ANOVA with Tukey’s correction was applied. *** and ** represent p values < 0.001, <0.01 respectively for WT vs cKO and ^^^^, ^^^ and ^^ represent p values < 0.0001, <0.001 and <0.01 respectively for WT vs cHET. n = 27 (WT control), 38 (*Tfap2d* Het), 26 (*Tfap2d* KO). **d**) Representative image of the mPFC showing immunostaining for FOS performed on the WT control, *Tfap2d*-cHet and *Tfap2d*-cKO brains isolated after 60-90 min of cued memory test. **e**) Graph depicting the number of FOS positive cells per mm^2^ detected within the different brain regions of the WT control, *Tfap2d*-cHet and *Tfap2d*-cKO brains isolated after 60-90 min of cued test. Mean ± s.e.m. depicted. Two-way ANOVA with Tukey’s correction was applied. ***, **, * represents p values < 0.001, <0.01, <0.05 respectively. For detailed statistics, please see Supplementary Table 19.

The BLC has a pivotal role in facilitating the learning of associations between neutral environmental cues and adverse outcomes, such as fear conditioning^1–3,5^. To test the effects of *Tfap2d*-driven BLC-related deficits on negative-association learning, we performed fear conditioning (Fig. 5a–c and Extended Data Fig. 14a–c). *Tfap2d*-KO and *Tfap2d*-cKO mice learned this association at the same rate as their respective heterozygous and wild-type groups (Fig. 5a and Extended Data Fig. 14a). During the contextual conditioning test, *Tfap2d*-Het mice spent significantly less time freezing compared with wild-type mice (Fig. 5b and Extended Data Fig. 14b). During the cued conditioning test, *Tfap2d*-KO and *Tfap2d*-cKO mice and their respective heterozygous controls displayed consistently higher levels of freezing compared with wild-type mice, indicating that loss or haploinsufficiency of *Tfap2d* causes alterations in the expression of learned threat responses (Fig. 5c and Extended Data Fig. 14c). Of note, mice that were heterozygous for *Tfap2d* displayed intermediate phenotypes, suggesting that limited *Tfap2d* expression was sufficient to mitigate the heightened cued fear freezing seen in *Tfap2d*-KO mice, but insufficient to restore behaviour to wild-type levels. Conversely, contextual freezing was decreased in *Tfap2d*-Het mice, suggesting that the regions involved in contextual freezing, hippocampal or PFC circuitry, were more affected by the reduction in *Tfap2d* dosage rather than its developmental loss, which may result in compensatory plasticity that masks the phenotype in *Tfap2d*-cKO mice. Similar phenomena have been observed in neuronal haploinsufficiency of other mouse knockout models of AP-2 family transcription factors^40^, although the functions of other AP-2 genes on BLC development are not known. Together, these results suggest that haploinsufficiency or loss of *Tfap2d* leads to nuanced changes associated with the interpretation of salient cues that may stem from alterations in BLC-related circuitry.

**Extended Data Fig. 15:**
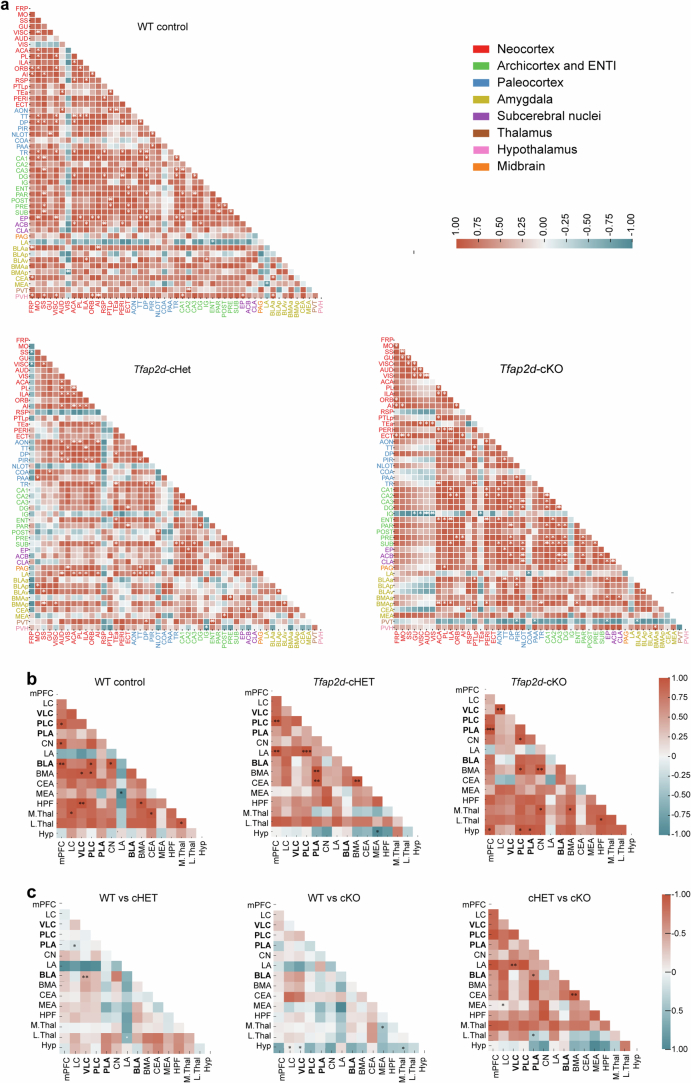
Alterations in functional connectivity following cued testing due to Tfap2d deficiency in the post mitotic excitatory neurons*.* **a)** Pearson correlation heatmap showing the functional connectivity between different brain regions measured by the FOS immunostaining of the WT control, *Tfap2d*-cHet and *Tfap2d*-cKO mice after the cued test. ***, **, * represents p values < 0.001, <0.01, <0.05, respectively (n = 4/genotype). Correlations were calculated using a two-tailed Pearson’s correlation test. For detailed statistics, please see Supplementary Table 10. **b)** Pearson correlation heatmap showing the functional connectivity between different brain regions measured by the FOS immunostaining of the WT control, *Tfap2d*-cHet and *Tfap2d*-cKO mice after the cued test. ***, **, * represents p values < 0.001, <0.01, <0.05, respectively (n = 4/genotype). Correlations were calculated using a two-tailed Pearson’s correlation test. **c)** Heatmap showing the differences seen in the Pearson correlations between WT controls, *Tfap2d*-cHet and *Tfap2d*-cKO calculated by Fisher’s z-transformations. ***, **, * represents p values < 0.001, <0.01, <0.05, respectively (n = 4/genotype). Highlighted in bold are the regions that express *Tfap2d*. Statistical significance of correlations was assessed using Fisher’s Z transformations, with a two-tailed test applied to compare correlation coefficients d. For abbreviations and details of regions grouped please refer to Supplementary Table 5. For detailed statistics, please see Supplementary Tables 20 and 21.

To discern the patterns of coordinated brain activity among multiple brain regions that are associated with fear expression during cued test^41^ and that may be impaired by *Tfap2d* haploinsufficiency or loss, we computed interregional correlations of FOS density, a measure of gross neuronal activity. We correlated FOS density between regions that are associated with cued fear such as the amygdala, neocortex, hippocampus and TH (Fig. 5d and Extended Data Fig. 15a–c). We found that significant correlations between cortical regions and the hippocampus formation in wild type mice were reduced or altered in *Tfap2d*-cHet and *Tfap2d*-cKO mice. In wild-type control mice, the FOS densities of LA and PL were negatively correlated, whereas this trend was absent in *Tfap2d*-cHet mice and remained insignificant in the *Tfap2d*-cKO mice (Fig. 5d and Extended Data Fig. 15a,b). Notably, there was a shift within the amygdala in the FOS correlation matrix for the *Tfap2d*-cKO mice compared with wild-type mice. In wild-type mice, FOS density in BLA was correlated with IL or mPFC; however, in *Tfap2d-*cKO mice, which were deficient in BLA, we observed a significant correlation in FOS density between the palaeocortical regions, including perirhinal and entorhinal cortex, and BMA with mPFC, which comprises PL, ILA and ACA (Fig. 5d and Extended Data Fig. 15a–c). These results align with the circuit alterations seen in the *Tfap2d*-cKO mice. However, these altered circuits do not seem to compensate for the loss of BLA, as evidenced by the altered functional connectivity and disrupted threat responses observed in the cued tests. Network graph analysis indicated a substantial reduction in the significant correlations among brain regions involved in threat response in *Tfap2d*-cHet mice, whereas in *Tfap2d*-cKO mice, we observed a shift in this connectivity compared with the wild type (Fig. 5d). These differences are consistent with the observed circuit deficits and indicate that *Tfap2d* haploinsufficiency alters functional connectivity of the threat-associated regions in a different manner to a total loss of *Tfap2d*.

Concurrently, we observed significantly fewer FOS-positive cells in the mPFC (ILA and PL) in *Tfap2d*-KO and *Tfap2d*-Het mice, as well as in *Tfap2d*-cKO and *Tfap2d*-cHet mice, compared with the respective wild-type groups (Fig. 5e and Extended Data Fig. 14d,e). We note that this pattern of FOS expression was not observed in the amygdala, where there were fewer FOS-positive cells in LA, BLAa and BLAp in *Tfap2d*-KO and *Tfap2d*-cKO mice compared with the respective heterozygous and wild-type mice. These data suggests that haploinsufficiency of *Tfap2d* in early life, although it is not sufficient to impair BLC formation, may nevertheless lead to differences in the expression of, but not the acquisition of, learned defensive behaviours, which are also reflected in differences in FOS levels across the brain.

## Discussion

This study advances our understanding of the development and evolution of the VLp. The key findings of this study are the identification of the SOX4-, SOX11- and TFAP2D-dependent gene regulatory network, which is essential for the proper development of VLp excitatory neurons. We highlight the nuanced role of TFAP2D gene dosage, which affects brain structure development, behaviour and connectivity. Although *Tfap2d* expression is conserved across species, its regulatory networks show key differences. The presence of an Alu cassette in the human *TFAP2D* locus suggests potential human-specific variations in its expression^42^, which may contribute to unique characteristics in these brain regions. These adaptations are likely to fine tune *Tfap2d* dosage, which is crucial for BLC development and connectivity.

These findings lay a foundation for future research on *Tfap2d*-dependent mechanisms, their association with neurodevelopmental disorders and the development of tools to selectively target specific brain regions. Mutations in *SOX4* and *SOX11* cause intellectual disability and other neurodevelopmental alterations^43–45^, and variants in the *TFAP2D* locus are associated with bipolar disorder and emotional dysregulation^46–50^. Our work thus opens new avenues for investigating the molecular mechanisms that underlie development of BLC and palaeocortex in the context of disease. This exploration may uncover how alterations in these processes contribute to neural traits and disease conditions and offer novel insights into pathogenesis.

## Methods

### Animals

All experiments were carried out with the protocol approved by the Committee on Animal Research at Yale University. The research methodologies for the use of mice (*Mus musculus*) and rhesus macaques (*Macaca mulatta*) were implemented following the guidelines sanctioned by the Yale University Institutional Animal Care and Use Committee (IACUC) and the directives of the US National Institutes of Health. The care and management of the animals were conducted within the precincts of the Yale Animal Resource Center, ensuring controlled environments for both prenatal and postnatal development stages of mouse and primate specimens. The mice were group-housed, maintaining a density of fewer than five individuals per cage, under environmental conditions regulated at 25 °C and 56% relative humidity, complemented by a photoperiod consisting of 12 h of light and 12 h of darkness. Nutritional needs were met ad libitum, coupled with veterinary oversight provided by the centre’s staff. The genetic lineage of the subjects was maintained on a C57BL/6 J strain, with experimental cohorts comprising both genders, randomly designated to respective studies. The detection of a vaginal plug in the mouse subjects was recorded as gestational day 0.5, marking the initiation of the experimental timeline. The *Sox4*^*fl/fl*^ and *Sox11*^*fl/fl*^ mice were a kind gift from V. Lefebvre. The *Tfap2d*-Lacz mice were a kind gift from M. Moser and the generation and genotyping were previously described^30^ (Extended Data Fig. 7a). These mice were generated by inserting a *Lacz* cassette that disrupts the exon 1 of the *Tfap2d* locus, abolishing the proper expression of the trapped allele, *Tfap2d*. The *Nex1-Cre* (*Neurod6-cre*) were a kind gift from the laboratory of K.-A. Nave and *CAT-Gfp* mice were procured from Jackson Laboratories^52^. We bred these mice with *Sox4*^*fl/fl*^ and *Sox11*^*fl/fl*^ to obtain controls (*Sox4*^*fl/+*^*;* *Sox11*^*fl/+*^*;* *Neurod6-cre;* *CAT-Gfp* or *Sox4*^*fl/fl*^*;* *Sox11*^*fl/fl*^), *Sox4*-cKO (*Sox4*^*fl/fl*^*; Sox11*^*fl/+*^*;* *Neurod6-cre*; *CAT-Gfp*), *Sox11*-cKO (*Sox4*^*fl/+*^*;* *Sox11*^*fl/fl*^*;* *Neurod6-cre;* *CAT-Gfp*) and *Sox4/Sox11*-cdKO (*Sox4*^*fl/fl*^*;* *Sox11*^*fl/fl*^*;* *Neurod6-cre;* *CAT-Gfp*). Further we also bred *Tfap2d*^*fl/fl*^ and obtained wild-type control (*Tfap2d*^+/+^*;* *Neurod6-cre;* *CAG*-*CAT-Gfp* or *Tfap2d*^*fl/fl*^) *Tfap2d*-cHet (*Tfap2d*^*fl/+*^*; Neurod6-cre;* *CAG*-*CAT-Gfp*) and *Tfap2d*-cKO (*Tfap2dfl/fl; Neurod6-cre;* *CAG*-*CAT-Gfp*). Refer to Supplementary Table 3 for genotyping details. Although blinding was not relevant for the primary mutant versus control comparison, other aspects of the study required careful design to minimize bias. Randomization was implemented while acquiring the data, including the behaviour data. Littermates (WT, HET and KO) were housed together, to avoid confounding housing effects on statistical analyses. The experimental cohort comprised male and female littermates aged between PD 120 and 180. Including samples from multiple litters further enhanced reproducibility.

### Postmortem human and macaque brain tissue

Human brain samples were collected postmortem at PCW17. Rhesus macaque brain samples were collected postmortem at PCD105. Whole slabs or whole hemispheres were post-fixed in 4% paraformaldehyde (PFA) for 48 h and then cryoprotected in an ascending gradient of sucrose (10%, 20%, 30%). Tissue was handled in accordance with ethical guidelines and regulations for the research use of human brain tissue set forth by the NIH (http://bioethics.od.nih.gov/humantissue.html) and the World Medical Association Declaration of Helsinki (http://www.wma.net/en/30publications/10policies/b3/index.html). All experiments using non-human primates were carried out in accordance with a protocol approved by Yale University’s Committee on Animal Research and NIH guidelines.

### Generation of *Tfap2d*-floxed mice

Guide RNA sequences (gRNAs) to insert *flox* sites were designed using an online program (http://zlab.bio/guide-design-resources)^53^. gRNAs with the minimum off-target effects were selected, and DNA oligonucleotides carrying guide RNA sequences were cloned into pX330 vector. The sequences of the guide oligonucleotides used are listed in Supplementary Table 3. Cas9 mRNA were in vitro transcribed from vector px330 linearized by restriction enzyme NotI-HF (New England Biolabs, R3189S) and purified by phenol/chloroform extraction. We inserted the flox sites into intron 1 and intron 3. Founders were genotyped and bred at least 3 generations to exclude chimeras. Genotyping of mice for floxed allele was performed using primers listed in Extended Data Fig. 8a,b, Supplementary Fig. 1 and Supplementary Table 3.

### In situ hybridization

The cDNA (*Tfap2d* human: ENSG00000008197; *Tfap2d* mouse:; *Tfap2d* macaque: ENSMMUT00000004057; *Lmo3*: ENSMUSG00000030226; *Etv1*: ENSMUST00000095767; *Mef2c*: ENSMUST00000163888, *Cyp26b1*: ENSMUST00000077705) for generating the human and mice probes was procured from Dharmacon and the plasmid was cut using NotI-HF enzyme. For the *Tfap2d* mouse probe, three different probes were amplified from PD0 and adult cDNA using the primers listed in Supplementary Table 3. The *Tfap2d* V3 probe worked best across ages and was used in the manuscript for detection of *Tfap2d* expression. The macaque probe was generated using respective neocortical tissues cDNA as a template by TA-cloning kit (Invitrogen, K202020). The cDNA clones were purified through the Qiagen clean up kit (Qiagen, 28104) and in vitro transcription (Millipore Sigma- Roche, 10999644001) was performed per the manufacturer’s instructions. Templates were purified by phenol/chloroform extraction and digoxigenin-labelled probes were synthesized using T3 (Roche, RPOLT3-RO) and T7 RNA polymerases (Roche, RPOLT7-RO) respectively, and RNA labelling mix (Roche, 11277073910) according to the manufacturer’s instructions. Probes were purified by ethanol precipitation, quantified, quality controlled and stored at −80 °C until hybridization. For in situ hybridization, slide-mounted cryo-sections at 60–70 μm thickness were processed. In brief, brains were fixed overnight at 4 °C in 4% PFA (Electron Microscopy Sciences, 15711) diluted in Dulbecco’s phosphate buffer (DPBS) (Thermo Fisher Scientific, 14190144), equilibrated for 12 h at 4 °C in 10% sucrose, and another 12 h at 4 °C in 30% sucrose in DPBS. Fixed brains were then embedded in OCT (Scigen, 23-730-571) and sliced on a cryostat (Leica Biosystem, CM1800). Slides were stored at −80 °C until processed for in situ hybridization. Sections were first post-fixed in 4% PFA in PBS for 15 min at room temperature, washed with PBS, and treated with 0.5 μg ml^−1^ proteinase K solution (15 min at room temperature for P0 and 30 min at room temperature for adults). Slides were post-fixed in 4% PFA in PBS for 15 min at room temperature, washed with PBS. Slides were treated with acetic acid and triethonalamine solution for 10 min, followed by PBS washes. Slides were then submerged in hybridization buffer (5× SSC, 50% formamide, 20% SDS and 250 μg ml^−1^ of Torula yeast RNA, aBSA (25 mg ml^−1^), heparin stock (50 mg ml^−1^)) supplemented with 1,000 ng ml^−1^ of the appropriate digoxigenin-labelled probe at 70 °C overnight. Sections were washed 3 times for 45 min at 70 °C in 2× SSC, 50% formamide, 1% SDS, and then three times with TBST and incubated overnight at 4 °C with an anti-digoxigenin antibody conjugated to alkaline phosphatase (1:5,000, Roche, 11093274910). Sections were washed and then rinsed in the substrate buffer (100 mM Tris-Cl pH9.5, 100 mM NaCl, 50 mM MgCl_2_, 0.1% Tween-20) before being overlaid with NBT/BCIP substrate (Roche). Colour development was done at room temperature in the dark until the desired signal was reached. Finally, sections were rinsed in EDTA solution and DPBS, washed in water and mounted with VectaMount AQ Aqueous Mounting Medium (Vector Laboratories, H-5501-60).

### RNA in situ hybridization (RNAscope) analysis on mouse and chicken brain tissue

Brains from the embryonic chicken (PCD17) and mouse (PCD16.5) were fixed in 4% PFA overnight at 4 °C. They were sectioned at 20 μm and slides were stored in −80 °C until use. RNAscope was performed as per RNAscope Multiplex FL v2 protocol and kit (323270) from ACD (Advanced Cell Diagnostics bio) with slight modification. In brief, the slides were thawed and washed with 1× PBS for 5 min at room temperature, followed by incubation at 60 °C for 30 min in the HybEZ hybridization system (321711). Slides were fixed for 30 min at 4 °C, followed by dehydration with ethanol (50%, 70%, and 100%, each for 5 min). Slides were then air dried for 5 min and then incubated at 60 °C for 15 min. Slides were treated with hydrogen peroxide for 10 min and washed with distilled water twice for 2 min. They were again incubated at 60 °C for 15 min and then washed with boiling water for 10 s. Further, target retrieval was performed using the RNAscope target retrieval reagents for 5 min in a boiling beaker. The samples were washed with distilled water again and then incubated with 100% ethanol for 5 min. Slides were incubated at 60 °C for 15 min. Using a barrier pen a boundary was created and the slides are incubated with 5 drops of the RNAscope protease III for 30 min. Samples are then washed with distilled water and incubated with the C1, C2, C3 probes for 2 h at 40 °C. After that samples were incubated sequentially with RNAscope Multiplex FL v2 Amp 1, Amp 2 and Amp 3 for 30 min each with intermittent washes with 1× wash buffer twice for 2 min. The slides were then incubated with horseradish peroxidase–C1, followed by tyramide signal amplification, horseradish peroxidase blocker each with intermittent washes with 1× wash buffer twice for 2 min. These steps were repeated for C2, C3 and slides were mounting. Slides were imaged on the VS 200 microscope (Olympus Microscopy). For mice tissue, milder conditions were used. We used 1 h fixation, 2 min target retrieval treatment and proteinase plus 10 min. We used the following probes: chicken *Tfap2d* (NPR-0050976, ACD) and mouse *Tbr1* (413301, ACD), *Ngn2* (417291, ACD) and *Sema5a* (508091, ACD).

### Immunohistochemistry

Brains isolated from prenatal and early postnatal mice were fixed overnight in paraformaldehyde (PFA) at 4 °C. Immunostaining was performed on the sections of 60–70 μm thickness cut on vibratome. Sections were blocked for 1 h at room temperature with blocking buffer consisting of 10% Donkey serum (Jackson ImmunoResearch, AB_2337258), 1% BSA (Millipore Sigma, A4612), 0.3% Triton X-100 (ThermoFisher, A16046.AP) in 1× PBS. After blocking, sections were incubated overnight at 4 °C with primary antibodies for SATB1 (1:500, Santacruz, sc-5989), TBR1 (1:1,000, Abcam, ab183032), NR4A2 (1:500, R&D systems, AF2156), CUX1 (1:1,000, Santacruz, sc-13024), BCL11B (1:2,000, Abcam, ab18465), FOS (1:500, Cell Signalling, 2250), BHLHE22 (1:1,000; Abcam, ab204791), GFP (1:500; Abcam, ab13970) and RFP (1:500, Abcam, ab124754). Sections were washed with washing buffer (0.3% Triton X-100 in 1× PBS) 3 times, 10 min each and were incubated with secondary antibodies (Jackson ImmunoResearch) for 1 h at room temperature, followed by 3 washes with washing buffer for 10 min each. Sections were mounted onto glass slides with vector shield (Vector labs, H-1000) and sealed with nail polish. All the slides were stored at −20 °C for further analyses. Antigen retrieval was performed on the brain sections prior to NR4A2 and TBR1 immunostaining (Dako Antigen Retrieval Solution, GV80511-2). For acquiring the images, LSM 800 (Zeiss Microsystems) and VS 200 microscope (Olympus Microscopy) were used. Analyses of the images was done using ZEN software, Qupath^54^, OlyVIS or Fiji^55^ using BioFormats plugin.

### In utero electroporation

In utero electroporation was performed on PCD12.5 *Sox4*^*fl/fl*^*;* *Sox11*^*fl/fl*^ timed-pregnant females with pNeurod1-cre and pCalnl-Tfap2d-Gfp on the right-side uterine horns and with pNeurod1-cre and pCalnl-Gfp into the left side uterine horns. Nearly 50% of all the embryos were injected with 0.5 μl DNA preparation (4 μg μl^−1^ DNA mixed with 0.05% Fast Green FCF dye (Sigma-Aldrich)). The DNA was injected into the lateral ventricle of the embryos and guided for electroporation to the pallial–subpallial boundary using gene paddle electrode (EC1 45-0122 3 × 5 mm, Harvard apparatus) and square-wave pulse electroporator (Harvard Apparatus) at 30–33 V, 5 pulses, 50 ms ON and 950 ms OFF At PCD16.5, viability of the electroporated pups was screened and the embryos in healthy conditions were used for further analysis. GFP expression was grossly determined by NIGHTSEA Full System with UV (EMS, SFA-UV). Embryos with the fluorescence signal were dissected to isolated brains and were fixed for 24 h in 4% PFA overnight at 4 °C, and brains were analysed as previously described. The brains were sectioned at 70 µm into 5 wells of a 24-well plate serially and all the sections from 1 well were stained for GFP, CC3 (1:1,000, Cell Signaling (Asp175) Antibody 9661), which marks dying cells and ADGRE1 (1:500, Bio-Rad, MCA497RT), which labels infiltration and activation of microglia^56^, using the protocol described above. For image acquisition, LSM 800 (Zeiss Microsystems) and VS 200 microscope (Olympus Microscopy) were used. Image analysis was done using ZEN software, Qupath^54^, OlyVIS or Fiji^55^ using the BioFormats plugin.

### Quantification of RNAscope and immunostaining

Images were acquired using the VS 200 microscope and analysed with QuPath. The images were opened directly in QuPath, and regions of interest (ROIs) were outlined using the Allen Brain Atlas or Mouse Developing Brain Atlas as references. For quantification, 5–6 coronal sections per brain, spanning from the anterior to posterior axis, were analysed. As we collected 70-µm sections into 5 wells, the sections analysed were nearly 400 µm apart. For RNAscope, the brains were sectioned at 20 µm and collected onto 10 slides. The number of cells above a set threshold in their respective channel and DAPI-positive cells within the ROI were quantified in QuPath and their ratio to DAPI was then calculated. Data from mutant brains were normalized to the wild-type littermates for CC3 and ADGRE1. For the quantification of BHLHE22 intensity, regions were analysed using the straighten plugin in ImageJ. A custom-made ImageJ program was used to divide the straightened PIR into 200 equal bins. The BHLHE22 intensity distribution was determined by calculating the intensity per bin relative to the total intensity across all bins. The data were plotted into graphs using GraphPad Prism version 10.0.0 for Windows (GraphPad Software). For statistics, the two-way ANOVA, one-way ANOVA or pairwise comparisons were directly performed in the GraphPad Prism.

### Generation and analysis of RNA-seq data

The cerebral cortex and amygdala from P0 *Sox4/Sox11*-cdKO, *Sox11*-cKO, *Sox4*-cKO and littermate control pups were collected (*n* = 3). Total mRNA was extracted using mirVANA Kit and DNAse digestion was performed using Tubro DNAse. Libraries were prepared with Illumina TruSeq mRNA preparation kit (Illumina RS-122-2101) for whole cortices, per the manufacturer’s instructions. Libraries were quality controlled by Tapestation/Bioanalyzer analysis and sequenced on the Illumina HiSeq 2000 platform at Yale Center for Genome Analysis (YCGA) to generate 75 bp single reads. Sequencing data were quality controlled by FastQC and aligned to the mouse genome (NCBI37/mm9) using STAR (v2.4.0e) (10.1093/bioinformatics/bts635). To improve the mapping quality of splice junction reads, mouse gene annotation retrieved from the GENCODE project (version M1) was additionally provided (10.1101/gr.135350.111). The command line “–sjdbOverhang 74” was used to construct a splice junction library. At least ten million uniquely mapped reads were obtained for each sample. Differential gene expression DEX analysis was performed by the R package DESeq2 (10.1186/gb-2010-11-10-r106) and principal components analysis was performed by the R package prcomp. Genes with |log_2_ fold change| ≥ 0.5 and a false discovery rate < 0.01 were classified as DEX. Integrated analysis was performed to identify the number of common or distinct DEX between *Sox4/Sox11*-cdKO, *Sox11*-cKO and *Sox4*-cKO. In total, 928 uniquely upregulated and 659 uniquely downregulated in *Sox4/Sox11*-cdKO were selected as candidate downstream targets, as shown in Supplementary Tables 1 and 2.

We further searched for cell types of amygdala using the public scRNA-seq dataset^36^. To find cell-type marker genes, we used the Seurat FindMarkers function. We performed the Wilcoxon rank sum test, and considered genes with a minimum expression ratio of 0.2, adjusted *P* value less than 0.05, logfc.threshold greater than 0.25, expression percentages less than 0.2 in other subtypes, and a fold change of expression percentages greater than 1.5 between the Excit_B subtype and the second subtype where the genes are detected as marker genes.

### Human gene expression analysis utilizing the Allen Brain Atlas and the BrainSpan Atlas

The images of brain structures presented in Fig. 2d were generated using the Freesurfer software^57^. To identify cortical regions, we used the Desikan–Killiany atlas, a commonly used atlas for human brain mapping^58^. *TFAP2D* expression values from two probes (A_23_P386973, CUST_2289_PI416261804) were taken from the Allen Brain Atlas^34^ (adult data) and summed. Then, the average of log_2_ expression values across six distinct individuals was computed. To visualize the expression levels of the gene, plot data, and to establish correspondence between the Desikan–Killiany atlas and the Allen Brain Atlas probes, the methods described in a previous study were used^51^. Subcortical regions were visualized using data from the Allen Brain Atlas website (http://atlas.brain-map.org). A standardized log_2_ gene expression colour bar graph across both cortical and subcortical regions was created using a custom R script developed in-house. To generate the graphs in Fig. 2c (Allen Brain Atlas adult data^34^) and Extended Data Fig. 4a (BrainSpan Atlas^35^ midfetal data; www.brainspan.org), the log_2_ *TFAP2D* expression values of the two different probes were averaged within a sample. For Fig. 2c, top-level structure names were changed to follow cortical cytoarchitectonics designations.

### Comparative analysis of *Tfap2d* gene expression across mammalian and non-mammalian species

To assess the expression patterns of *TFAP2D* across different species, we reanalysed public single-nucleus transcriptome datasets for amygdala in human (*Homo sapiens*), macaque (*M. mulatta*), mouse (*M. musculus*) and chicken (*Gallus gallus*). We checked the expression level of *Tfap2d*, *Sox4*, *Sox11*, *Lmo3*, *Etv1* and *Mef2c* across major cell types, space clusters and cell-type clusters, as identified by the original paper^38^. For cross-species comparison, we only included the excitatory neurons.

### Analysis of *Tfap2d* regulatory regions and SOX11 ChIP–seq data

To determine what regions within the *Tfap2d* locus can be potential enhancers, we re-processed a single-cell ATAC-seq embryonic dataset of the mouse cortex^33^ using Signac version 1.1.0 (https://satijalab.org/signac/), to obtain ATAC-seq peaks that are accessible in migrating excitatory neurons in PCD13.5 onwards. We further refined the list of putative peaks by requiring that they intersect with occurrences of *Sox4* or *Sox11* JASPAR motifs (MA0867.1, MA0867.2, MA0869.1, MA0869.2). Similarly to regions directly bound by *SOX4* and *SOX11* within *Tfap2d* locus, we analysed data generated from ChIP–seq using an antibody against H3K27ac, a histone mark enriched in promoters and enhancers^32^. The *SOX11* ChIP–seq data were directly accessed from Bergsland et al.^29^.

### Chromatin immunoprecipitation and rtPCR

Cortices from PCD15.5 from wild-type and *Sox11*-cKO mice were isolated and were crosslinked with a formaldehyde solution at a final concentration of 1% at room temperature for 10 min. l-Glycine (AmericanBio) at a final concentration of 125 mM was added and incubated for 5 min at room temperature to quench the cross-linking. The tissue was spun down and lysed in the hypotonic solution (50 mM Tris-Cl pH 7.5, 0.5% NP40, 0.25% sodium deoxycholate, 0.1% SDS, 150 mM NaCl) on ice for 10 min to obtain the nuclei. Nuclei were centrifuged at 600*g* for 5 min at 4 °C and pellets were resuspended in the SDS lysis buffer (1% SDS, 10 mM EDTA and 50 mM Tris-Cl, pH 8.1) prior to being sheared into 200–500 bp size fragments using a sonicator (M220 Focused-ultrasonicator, Covaris). The sheared DNA was diluted with the ChIP dilution buffer (0.01% SDS, 1.1% Triton X- 100, 1.2 mM EDTA, 16.7 mM Tris-Cl, pH 8.1, 167 mM NaCl) and was precleared with magnetic protein A/G beads (Thermo Scientific) for 1 h at 4 °C. For ChIP, 10 μg anti-*SOX11* (Abcam, ab229185), 1 μg Pol II (Sigma-Aldrich, 05-623) and 10 μg of IgG (Diagenode, C15410206) were used. Samples were incubated on constant rotation overnight at 4 °C. Magnetic protein A/G beads (EMD Millipore,166-63) were blocked with 1 mg ml^−1^ bovine serum albumin (BSA) (Sigma-Aldrich) and tRNAs were added to the chromatin-antibody complexes for 4 h at 4 °C. Beads were washed with low salt (0.1% SDS, 1% Triton X-100, 2 mM EDTA, 20 mM Tris-Cl, pH 8.1, 150 mM NaCl), high salt (0.1% SDS, 1% Triton X-100, 2 mM EDTA, 20 mM Tris-Cl, pH 8.1, 500 mM NaCl), LiCl (0.25 M LiCl, 1% IGEPAL CA630, 1% deoxycholic acid (sodium salt), 1 mM EDTA, 10 mM Tris-Cl, pH 8.1) and TE (AmericanBio), sequentially for 3 min each. ChIP DNA was incubated overnight at 65 °C for reverse cross-linking and subjected to RNAse A treatment (37 °C, 1 h) and proteinase K treatment (55 °C, 2 h), then purified on PCR purification columns. For input control, 5 μg crosslinked chromatin from each sample was also treated by reverse cross-linking, RNAse A (Thermo Scientific) and Proteinase K (Millipore Sigma) together with IP samples and purified by PCR purification columns. DNA amounts were quantified by PicoGreen assay (Thermo Scientific). The samples were eluted in 20 μl of TE. Each sample is diluted 1:5 and processed for rtPCR using the Bio-Rad SYBR Green Mix and Bio-Rad machine. Samples were run in triplicates and fold enrichment was calculated over the input. Two-way repeated measures ANOVA with Tukey’s multiple comparison correction was applied.

#### Enhancer conservation

We used mafsInRegion^59^ from UCSC tools to obtain MULTIZ60 alignments for each selected enhancer. We stitched maf alignments and converted them to fasta using the script maf_to_concat_fasta.py from bx-python (bx-python). Pairwise alignment distance between species of each of the selected H3K27ac peaks were obtained using the function dist. alignment from the seqinr package in R.

### Luciferase assays

For luciferase reporter plasmids, mouse *Tfap2d* enhancer candidate fragments (E1–E6) were amplified by PCR from genomic DNA and inserted into the pGL4.24 vector (E8421, Promega). Mutant form of each enhancer lacking *SOX4* or *SOX11* binding sites were also generated. The sequences of the PCR primers and PCR products are listed in Supplementary Table 3. The Neuro2a mouse neuroblastoma cell line was purchased from ATCC. The cell line was authenticated by morphology or genotyping, and no commonly misidentified lines were used. All lines tested negative for mycoplasma contamination, checked monthly using the MycoAlert Mycoplasma Detection Kit (Lonza). Luciferase assay was performed with Neuro2a cells basically as described^60^. Neuro2a cells were transfected using Lipofectamine 3000 (L3000015, Thermo Fisher Scientific) with either mouse pCagig-Sox4−3×Flag, pCagig-Sox11−3×Flag or empty pCAGIG (Addgene #11159), together with one of the pGL4.24 luciferase vectors generated with enhancer sequences as described above. pGL4.73 The *Renilla* luciferase plasmid (E6911, Promega) was co-transfected as a control transfection efficiency. The luciferase assays were performed 48 h after transfection using the Dual-Luciferase Reporter Assay System (E1910, Promega) according to the manufacturer’s instructions. For each sample, three replicates were measured in a single assay. Luciferase activity was measured and quantified by GloMax Explorer (GM3500, Promega). The values for firefly luciferase/*Renillia* ratio for *Sox11* or *Sox4* were normalized to their respective pCagig controls.

### Nissl staining

Perfused brains were sectioned at 40 μm thickness and mounted and dried on slides. A 1:12 series through the brain was stained for Nissl. In brief, sections went through a demyelination step including first an ascending series of ethanol followed by a descending series of ethanol, stained with cresyl violet acetate stain for 5 min and then briefly washed with water and ethanol. Sections were destained using a mild differentiating solution (50% ethanol + 5 drops of glacial acetic acid) and further dehydrated in an ascending series of ethanol before being placed in xylene and coverslipped. Slides were scanned with the Leica Aperio digital slide scanner at 20× magnification. Samples were observed for gross morphological alterations due to genotype.

### Acetylcholinerase histochemistry and stereological analysis

For each animal, a 1:12 series of sections through the brain was stained with acetylcholinerase^61^, which demarcates the LA from the BLA. Each series contained between four and six sections through the anterior–posterior extent of the amygdala. Slides were scanned with the Leica Aperio digital slide scanner (Leica) at 20× magnification. Images used for stereological estimates were cropped using Leica’s Aperio ImageScope software and saved as JPEG. The Cavalieri estimator probe, implemented in Stereoinvestigator (MBF Bioscience) was used to calculate BLA and La volumes using a grid size of 10 μm. Volume estimates used are corrected for overprojection.

### Diffusion tensor imaging

Nine postmortem mouse brains (3 wild-type control, 3 *Tfap2d*-Het and 3 *Tfap2d*-KO) were perfusion-fixed in with 4% paraformaldehyde solution in 0.1 M PBS for 24 h. Following perfusion–fixation, mouse brains were immersed and stored in 0.1 M PBS for 24 h and were transferred to Fomblin (SPI Supplies 69991-67-9) just before imaging. Diffusion MRI was acquired on a BioSpin 9.4 T MRI (Bruker) machine for each subject using a 3D echo-planner-imaging diffusion sequence (*b* = 1,500 s mm^−2^; 30 directions) with the following parameters^62^: repetition time = 1,250 ms; echo time = 26 ms. The resolution was 0.1 mm isotropic. Overall scanning time was 19.5 h.

### DTI image processing and tractography

Cerebral cortical and subcortical ROI and TH were manually defined according to Paxinos^63^ and the Allen Mouse Brain Atlas^64^ (https://mouse.brain-map.org/static/atlas) by D.A., N. Salla and N.K. without prior knowledge of the experimental groups. Whole-brain deterministic tractography^65–67^ was performed with Diffusion Toolkit (Version 0.6.4.1) software with a fractional anisotropy threshold of 0.02 and an angular threshold of 60 degrees. Fibres that pass through each pair of ROIs were delineated and quantified. Visualization of the tracts and mouse brain was generated using MRtrix361 software^68^ (v.3.0.0-65-g91788533). Circular visualization of the tractography derived connectivity between brain regions was generated using Circos software package^69^.

### Retrograde and anterograde neuronal tracing

To determine the effects of BLC deficits associated with *Tfap2d* loss and confirm the DTI results, we performed the AAV-based tracing from the mPFC, with retrograde and anterograde tracers injected simultaneously into the mPFC of PD120–PD180 mice. In brief, mice were anaesthetized by injecting a ketamine/xylazine solution and head fixed in a stereotactic frame. Thirty minutes before surgery, buprenorphine was administered. After lubricating the eyes and shaving the fur, an incision of <1 mm was made. A craniotomy was made with a round 0.5-mm drill bit at the desired coordinates (for the mPFC: medial–lateral ±0.35, anterior–posterior 2.0, dorsal–ventral 2.5). Using a Hamilton neuros syringe (0.5 ml), we mixed and injected 100 nl of each of AAVrg-CAG-Tdt (59462-AAVrg, Addgene) of AAV1-Camk2a-eGfp (50469-AAV1, Addgene) into the mPFC. To prevent the virus from spreading along the injection tract, the needle was held in place for at least 10 min. After injections, the skin was sutured, and the mice were returned to the cage. Approximately 3 weeks later, the mice were euthanized, and their brains were collected. The brains were coronally sectioned on a vibratome to obtain 70 µm thick sections. After staining the sections with anti-GFP antibody (1:500; Abcam, ab13970) and anti-RFP antibodies (1:500, Abcam, ab124754), the sections were imaged with VS 200 (Olympus Microscopy). The images were analysed using Qupath software^54^ where the outline for the ROI was created and the number of cells projecting to mPFC were counted above threshold and the intensity of the axons in each region was measured using the ImageJ plugin.

### Mouse behavioural assessments

We performed a series of tests on the mice to determine their exploratory, innate or learned anxiety-related behaviours^70,71^. The experimental cohort comprised of male and female littermates aged between PD120 and PD180. Mice were acclimatized in the room for 30 min to 1 h before the beginning of any behaviour experiments. The experiment began with the apparatus being sanitized using 70% ethanol. In the following trials, water was used for cleaning instead. The first trial featured control mice and to mitigate the strong ethanol scent that might alter their behaviour, cage dust was rubbed on the apparatus. The results from this initial trial were omitted from the final analysis. The tests were performed in the order listed below with a spacing of 24 to 48 h between tests.

#### Open-field test

The rectangular arena (50 × 50 cm) was cleaned, and using ethovision software it was divided into centre, border and wall zones. Mice were placed in the centre of the open-field arena to allow them to explore the open-field area for 20 min. The activity of the mouse was videographed using Noldus Ethovision XT software. The EthoVision software automatically records the movement of the mouse, tracking the time spent in the centre versus the border zones, as well as the total distance moved and the speed of movement. The collected data were analysed to determine the preference of the mouse for the centre or border of the arena. Less time spent in the centre and more in the border indicates higher anxiety-like behaviour. Data are presented as mean ± s.e.m. Statistical analyses involved two-way ANOVA with Tukey’s multiple comparison correction, utilizing GraphPad Prism software (version 9). *n* = 16 (wild-type control), 33 (*Tfap2d*-Het), 29(*Tfap2d*-KO), 14 (wild-type control), 14 (*Tfap2d*-cHet) and 15 (*Tfap2d*-cKO).

#### O-maze

The elevated O-maze is clean and positioned in a quiet room and the EthoVision system was calibrated to recognize the different sections of the maze (open arms versus closed arms). Mice were gently placed in the centre of the closed arm of O-maze and allowed to explore freely for 10 min. The EthoVision software tracked movements, specifically recording entries into and the time spent in the open and closed arms. The data were analysed to assess the preference of the mouse for closed arms over the open arms, which indicates anxiety-like behaviour. The frequency of entries and the duration spent in each arm type are key metrics for this analysis. Statistical analyses involved two-way ANOVA with Tukey’s multiple comparison correction, utilizing GraphPad Prism software (version 9). *n* = 14 (wild-type control), 38 (*Tfap2d*-Het), 27 (*Tfap2d*-KO), 19 (wild-type control), 13 (Tfap*2d*-cHet) and 16 (*Tfap2d*-cKO).

#### Light-dark box

The assessment of anxiety levels using the light/dark box test was conducted in accordance with the methodology detailed by Bourin et al.^72^. In summary, the experimental setup involved a dual-compartment apparatus, each compartment made of opaque Plexiglas and measuring 18 cm in length, 10 cm in width, and 13 cm in height. The light compartment was illuminated by a 100 W lamp positioned overhead, with the light diffused through a transparent Plexiglas lid. Mice were granted access between the two compartments via a small aperture connecting them. Mice were initially placed within the illuminated section. Observations commenced from the moment a mouse first entered the dark compartment, continuing for a duration of 5 min. The amount of time the mouse spent in the light box was noted. The mice were scored blindly without information about their genotypes. The primary behavioural metrics quantified included the duration of time spent within the light compartment, measured in seconds, and the frequency of compartment transitions. Data are presented as mean ± s.e.m. Statistical analyses involved one-way repeated measures ANOVA with Tukey’s multiple comparison correction, using GraphPad Prism software (version 9). *n* = 10 (wild-type control), 10 (*Tfap2d*-Het) and 7 (*Tfap2d*-KO).

#### Tail suspension

Depression-like behaviours were measured using tail suspension and forced swim tests^73,74^. Mice were suspended by the tail with a paper clip attached with adhesive tape about 5 mm from the end of the tail. Time spent immobile was recorded over the duration of 6 min. The video was recorded during the test. After completion of the test, mice were returned to a holding cage until all cage-mates were tested. After completion of the experiment, all mice were returned to the home cage and transferred back to the holding room. The videos were scored blindly by an independent person for the time mice were mobile and immobility time was calculated. Data are presented as mean ± s.e.m. Statistical analyses involved one-way repeated measures ANOVA with Tukey’s multiple comparison correction, utilizing GraphPad Prism software (version 9). *n* = 18 (wild-type control), 24 (*Tfap2d*-Het) and 13 (*Tfap2d*-KO).

#### Forced swim

Mice were gently introduced into transparent 4-l glass beakers filled with 2.5 l of water at ambient temperature. Each subject was gently introduced into the water, ensuring their ability to maintain their nostrils above the surface for breathing. On day 1 of the experimental procedure, mice were exposed for a duration of 15 min. Subsequently, on the following day, the immersion time was reduced to a series of 5 min. The videos of the test were recorded and were scored blindly by an independent person for the time mice were mobile and immobility time was calculated. Immobility was characterized by a state of passive floating, wherein the subject performed minimal movements necessary to remain afloat. Data are presented as mean ± s.e.m. Statistical analyses involved one-way repeated measures ANOVA with Tukey’s multiple comparison correction, using GraphPad Prism software (version 9). *n* = 20 (wild-type control), 23 (*Tfap2d*-Het) and 13 (*Tfap2d*-KO).

#### Fear conditioning

The experimental cohort comprised of male and female littermates’ siblings aged between PD120 and PD180. The method used for contextual and cued fear conditioning was outlined previously^75–78^, with some modifications. The experiment consisted of four parts: habituation, training, contextual memory assessment, and cued memory evaluation. During the habituation phase, mice were introduced to a dimly lit enclosure composed of dark plastic with a cardboard floor, incorporating sawdust from their original housing to establish context A, without the administration of shocks or cues. For the training phase, the subjects were relocated to a differently structured chamber equipped with illuminated stainless steel grids capable of delivering electric shocks, designated as context B, and infused with a cinnamon scent beneath the grids to serve as a unique olfactory stimulus. Following a 160-s acclimatization period, an auditory cue (75 decibels, 2.8 kHz) was emitted for 20 s, with the concluding 2 s overlapping with an electric shock (0.5 mA). The mice experienced 6 instances of tone-shock associations, interspersed with 40-s intervals, and concluded with a 60-s relaxation phase before being returned to their original cages. The subsequent day involved testing for contextual memory in the same chamber (context B) but with the introduction of a vanilla scent beneath the grids to differentiate from the training phase’s cinnamon odour, thereby focusing on contextual rather than olfactory memory recall. Cued memory was assessed in the original context A setup, using the same auditory cues as during training but omitting the shocks. All sessions were video recorded, and the freezing behaviour of the mouse—defined as the absence of movement for each 3-s video frame—was analysed. No significant variances in body weight or shock response were noted. Data are presented as mean ± s.e.m. Statistical analyses involved two-way ANOVA for the training and cued memory test and multiple comparisons with Tukey’s correction, using GraphPad Prism software (version 9). For conditioned memory test, one-way ANOVA with Tukey’s correction was applied. *n* = 27 (wild-type control), 38 (*Tfap2d*-Het), 26 (*Tfap2d*-KO), 16 (wild-type control), 12 (*Tfap2d*-cHet) and 16 (*Tfap2d*-cKO).

### FOS immunostaining and registration to the mouse brain common coordinate framework

To determine the activity of different brain regions after cued memory test, we performed a whole-mount FOS staining using the LifeCanvas Technologies. We could not utilize the extensively used, targeted recombination in active populations (TRAP) mice^79^ for these experiments because *Neurod6-cre* is expressed in the cerebral excitatory neurons of the conditional *Tfap2d* mice, leading to reporter activation during prenatal development, which occurs prior to FOS-driven Cre-ER(T2) expression. Mice were euthanized 60–90 min after the cued memory test using isofluorane followed by transcardial perfusion with ice-cold 1× PBS with 10 U ml^−1^ heparin until the fluid ran clear, followed by ice-cold 4% PFA. The extracted brains were incubated in 4% PFA solution at 4 °C for 24 h with gentle shaking. The samples were cleared and stained by LifeCanvas Technologies using their protocol^80^. Samples were cleared using a SHEILD OFF solution for 3 days at 4 °C, followed by incubation in the SHIELD ON for 24 h at 37 °C with light shaking. Samples were then processed for delipidation and staining using SmartBatch + . The brains after delipidation were blocked with donkey serum or 2 days, followed by primary antibody incubation for 1–3 days and secondary antibody for incubation 6 h. After each primary and secondary antibody incubations, stringent washes and PFA fixation was performed. The brains were imaged using SmartSPIM at 4 μm *z*-step and 1.8 μm *xy* pixel size. After imaging, the samples underwent automated atlas registration to the Allen Brain Atlas via LifeCanvas Technologies using rigid, affine, and b-spline warping algorithms provided by SimpleElastix, for both propidium iodide and FOS (647) channels. For the KO brains where the BLC shape was altered, the protocol was modified and manually registered for atlas registration. For cell detection, custom cell detection networks developed by LifeCanvas Technologies, employing a two-step approach with a U-Net architecture for candidate detection and a 3D ResNet for classification, and were trained on hand-tagged 3D ROIs to accurately identify cell locations. The detected cells were then mapped onto the Allen Brain Atlas for quantitative analysis. Colocalization analysis determined cell co-expression of FOS and propidium iodide by measuring the Euclidean distance between cells in different channels, with co-expressed cells within a 3-voxel distance threshold being counted for each atlas-defined brain region. The output generated would provide the number of FOS-positive cells per mm^3^ in each region labelled by the Allen Brain Atlas.

### Functional network construction

We developed a comprehensive analytical framework to examine the relationship between various regions functionally active after cued memory test based on the FOS expression^81,82^. The analysis was performed using Python, with key libraries including Pandas for data manipulation, Seaborn and Matplotlib for visualization, NetworkX for network analysis, and SciPy for statistical tests. We calculated Pearson correlation coefficients to assess the linear relationship between each pair of subregions^41,82^. To evaluate the statistical significance of these correlations, *P* values were computed. Heat maps were generated to visualize the correlation matrix amongst brain regions associated with threat responding, with annotations indicating the significance levels. The upper triangle of the matrix was masked to prevent redundancy, given the symmetric nature of correlation matrices. A diverging colour palette from blue (negative correlation) to red (positive correlation) was applied for visual clarity. Network graphs were created to visualize the connectivity between subregions based on their correlation coefficients. Edges were drawn for correlations surpassing a defined cutoff of 0.85, emphasizing stronger relationships. Edge colours transitioned from blue to red, representing the spectrum of correlation strengths. We compared the correlation coefficients between experimental groups (wild-type, Het and KO) using Fisher’s *z*-transformation followed by significance testing. First, Pearson correlation coefficients were computed for each group based on the averaged data across grouped regions. To stabilize variance and allow for the comparison of correlations between groups, we applied Fisher’s *z*-transformation to the correlation coefficients. We then calculated the *z*-score for the difference between the two *z*-transformed correlations across groups. The *P* value associated with the *z*-score was determined using the cumulative distribution function of the standard normal distribution, and statistical significance was annotated on heat maps of the correlation differences with significance thresholds. The data related to these experiments is available in Supplementary Table 10 and Supplementary Tables 20 and 21. Abbreviations are available in Supplementary Tables 4 and 5.

### Statistics and reproducibility

All data are reported as mean ± s.e.m. with data analysis being conducted using one-way and two-way ANOVA with Tukey’s post hoc multiple-comparisons adjustment or unpaired *t*-tests for comparisons between two groups. Fisher’s exact tests were also used and the details of statistics are presented in the Supplementary Tables 6–21. Details of the number of replicates in each experimental group together with appropriate statistical analyses are shown in the figure legends. For the representative images the numbers of biological replicates used are following. Figure 1a includes data from 3 brains per genotype for Nissl, *Lmo3*, *Etv1* and *Mef2c* staining, and 4 brains per genotype for GFP and SATB1. Figures. 1d and 3b,c use 4 brains/genotype. Figures 2b and 5f use 2 brains, and Figs. 2e and 5c include data from 3 brains per age. Extended Data Figs. 1a,b and 2a,c incorporate 4 brains per genotype. Extended Data Fig. 11b uses 15 or more samples per genotype. These animals were tested for recombination events for 5′ and 3′ sites of the *Tfap2d*-floxed allele across multiple generations. We did not observe recombination between them. Finally, Extended Data Fig. 11c and Fig. 12a–c use 3 brains per genotype. Significance threshold was set at *P*  <  0.05. All statistical analysis and plotting were performed in GraphPad 9 and 10 (GraphPad) or in Python. All of the figures were created using Adobe Illustrator (Adobe Systems).

### Reporting summary

Further information on research design is available in the Nature Portfolio Reporting Summary linked to this article.

## Online content

Any methods, additional references, Nature Portfolio reporting summaries, source data, extended data, supplementary information, acknowledgements, peer review information; details of author contributions and competing interests; and statements of data and code availability are available at 10.1038/s41586-024-08361-5.

## Supplementary information

Supplementary Information This file contains the Supplementary Discussion, Supplementary Fig. 1 and descriptions for Supplementary Tables 1–21 (tables supplied separately).

Reporting Summary

Supplementary Tables Supplementary Tables 1–21

## Data Availability

RNA-seq data were deposited into the NIH Bioproject under accession number PRJNA1150339.
