# Supplementary material for: Specification of claustro-amygdalar and palaeocortical neurons and circuits
Source: Nature. 2025 Jan 15;638(8050):469–78. doi: 10.1038/s41586-024-08361-5 (PMC11821539; doi:10.1038/s41586-024-08361-5)
Supplement: Supplementary file 1 — This file contains the Supplementary Discussion, Supplementary Fig. 1 and descriptions for Supplementary Tables 1–21 (tables supplied separately). [file 41586_2024_8361_MOESM1_ESM.pdf]

nature portfolio

https://doi.org/10.1038/s41586-024-08361-5

Supplementary information

Specification of claustro-amygdalar and palaeocortical neurons and circuits

In the format provided by the authors and unedited

Supplementary Information

Supplementary Text

Discussion

This study addresses a critical question at the juncture of neuroscience and evolutionary biology, exploring the molecular mechanisms that underpin the development and evolution of the ventrolateral pallium. Our analysis yields three principal findings. First, we present evidence demonstrating the indispensable role of the SOX4 and SOX11-dependent post-mitotic gene regulatory network in the proper development of ventrolateral pallial ExNs, particularly within the BLC complex. This network orchestrates the expression of genes with highly specific patterns within the developing claustro-amygdalar complex and paleocortex, which are crucial for neuronal development and connectivity. These insights build upon and extend the known functions of these TFs in neuronal specification [21] including broadly neocortical and archicortical ExNs [19]. Here, we highlight a specific regulatory node essential for the molecular specification of ventrolateral pallial ExN development.

Second, our findings identify TFAP2D as a pivotal TF within this ventrolateral pallial regulatory node. Its expression, highly specific to ventrolateral pallial ExNs and contingent upon SOX4 and SOX11 activity, including direct SOX11 binding at enhancer *E2* in the *Tfap2d* locus, underscores its critical role. This study significantly expands our understanding of TFAP2D's role beyond its previously documented expression in the human and non-human primate amygdala [23-26], revealing its broader pattern across paleocortical and the associated mesocortex. The nuanced role of TFAP2D is further evidenced by the delicate balance of its gene dosage, which profoundly affects the development of these structures and results in altered behaviors and connectivity patterns, particularly between the BLC and PFC.

Thirdly, we showed that while *Tfap2d* expression is conserved across species, its regulatory networks display key divergent features. Similarly dynamic evolutionary mechanisms have been proposed for other gene-TF associations[37]. These *Tfap2d*-associated enhancer adaptations likely regulate *Tfap2d* dosage, which prenatally regulates the BLC ExNs survival, and their migration to the

BLC, resulting proper BLC enlargement and postnatally regulates BLC- PFC connectivity and its proper functioning. Further, the presence of an Alu cassette in the human *TFAP2D* locus, absent in other primates [42], hints at potential human-specific variations in *TFAP2D* expression, a factor that could play a role in shaping our unique characteristics within these brain regions. Future studies involving generation of cross-species enhancer mice models are needed to explore additional SOX4 and SOX11-bound *Tfap2d* enhancers from other species to determine their functional consequences to regulate *Tfap2d* expression, its dosage, and their effects on BLC formation and connectivity.

These findings also emphasize the nuanced influence of *Tfap2d* gene dosage on developmental trajectories, BLC-PFC connectivity, and behaviors that resemble alterations observed in certain neurodevelopmental and neuropsychiatric disorders. The changes in functional connectivity seen in the PFC of *Tfap2d* cHET mice, which have an intact and active BLA, presents reassuring evidence of altered functional loss of PFC-BLC connectivity. Thus, the *Tfap2d* haploinsufficiency may not disrupt ventrolateral pallium including BLC but may alter gene expression that affects its connectivity to the PFC and therefore its function.

To our knowledge, the specific expression of *Tfap2d* in the ventrolateral pallial regions and the BLC loss phenotypes associated with its depletion are unique and novel aspects of our study. Furthermore, we have revealed a gene regulatory network underlying the development and connectivity of ventrolateral pallial ExN. Thus, this study lays the foundation for identifying additional features of *Tfap2d*-dependent mechanisms and components of this gene regulatory networks and their potential associations with neurodevelopmental disorders. This knowledge will also enable the development of tools to selectively target BLC, LCS, and PSB. Accordingly, mutations in SOX4 and SOX11 cause intellectual disability and other neurodevelopmental alterations [43-45], and variants within the TFAP2D locus are associated with bipolar disorder and emotional dysregulation [46-50], opening new avenues for investigating the molecular mechanisms underlying BLC and paleocortical development in the context of disease. This exploration may uncover how alterations in these

processes contribute to neural traits and disease conditions, offering novel insights into their pathogenesis.

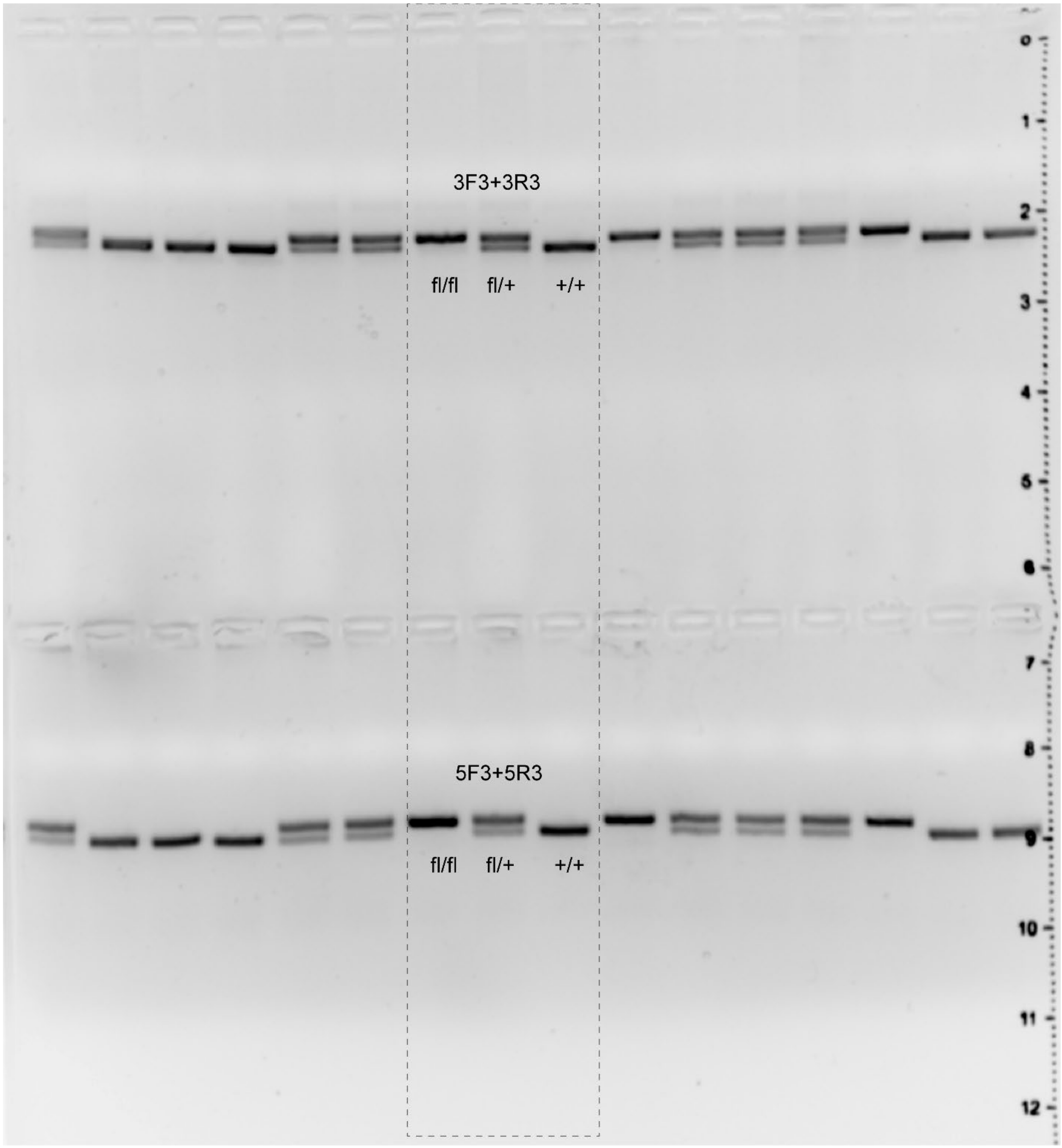

Supplementary Fig. 1: Original picture of the genotyping gel presented in Extended Data Fig. 10b

List of Tables

Supplementary Table 1. Genes with Increased Expression Exclusively in the *Sox4; Sox11* cdKO as Compared to *Sox4* cKO, *Sox11* cKO and WT controls

Supplementary Table 2. Genes with Decreased Expression Exclusively in the *Sox4; Sox11* cdKO as Compared to *Sox4* cKO, *Sox11* cKO and Controls

Supplementary Table 3. List of the Oligos and Primers

Supplementary Table 4. List of Abbreviations for Figure 5d-e and Extended Data Figure 14a

Supplementary Table 5. List of Abbreviation for Extended Data Figure 19

Supplementary Table 6. Statistics for Fig. 1e and Fig. 1f

Supplementary Table 7. Statistics Related to Figure 3d

Supplementary Table 8. Statistics for Fig. 4

Supplementary Table 9. Statistics for Fig. 5a-c

Supplementary Table 10. Pearson Correlation Values and Statistics for Fig. 5d and Extended Data Fig. 15a

Supplementary Table 11. Statistics for Fig. 5e

Supplementary Table 12. Statistics for Extended Data Fig.3

Supplementary Table 13. Statistics for Extended Data Fig. 4b

Supplementary Table 14. Statistics for Extended Data Fig. 9e

Supplementary Table 15. Statistics for Extended Data Fig. 11

Supplementary Table 16. Statistics for Extended Data Figure 12

Supplementary Table 17. Statistics for Extended Data Figure 13a-c

Supplementary Table 18. Statistics for Extended Data Figure 13g-k

Supplementary Table 19. Statistics for Extended Data Figure 14

Supplementary Table 20. Pearson's Correlation Values and Statistics for Fig. 15b

Supplementary Table 21. Fisher's Z Transformations and Statistics for Fig. 15c
