# Supplementary material for: Specification of claustro-amygdalar and palaeocortical neurons and circuits
Source: Nature. 2025 Jan 15;638(8050):469–78. doi: 10.1038/s41586-024-08361-5 (PMC11821539; doi:10.1038/s41586-024-08361-5)
Supplement: Supplementary file 2 — Reporting Summary [file 41586_2024_8361_MOESM2_ESM.pdf]

Corresponding author(s): Nenad Sestan

Last updated by author(s): 10-03-2024

Reporting Summary

Nature Portfolio wishes to improve the reproducibility of the work that we publish. This form provides structure for consistency and transparency in reporting. For further information on Nature Portfolio policies, see our Editorial Policies and the Editorial Policy Checklist.

Statistics

For all statistical analyses, confirm that the following items are present in the figure legend, table legend, main text, or Methods section.

n/a	Confirmed	
☐	☒	The exact sample size (*n*) for each experimental group/condition, given as a discrete number and unit of measurement
☐	☒	A statement on whether measurements were taken from distinct samples or whether the same sample was measured repeatedly
☐	☒	The statistical test(s) used AND whether they are one- or two-sided<br>*Only common tests should be described solely by name; describe more complex techniques in the Methods section.*
☒	☐	A description of all covariates tested
☐	☒	A description of any assumptions or corrections, such as tests of normality and adjustment for multiple comparisons
☐	☒	A full description of the statistical parameters including central tendency (e.g. means) or other basic estimates (e.g. regression coefficient) AND variation (e.g. standard deviation) or associated estimates of uncertainty (e.g. confidence intervals)
☐	☒	For null hypothesis testing, the test statistic (e.g. *F*, *t*, *r*) with confidence intervals, effect sizes, degrees of freedom and *P* value noted<br>*Give P values as exact values whenever suitable.*
☒	☐	For Bayesian analysis, information on the choice of priors and Markov chain Monte Carlo settings
☒	☐	For hierarchical and complex designs, identification of the appropriate level for tests and full reporting of outcomes
☐	☒	Estimates of effect sizes (e.g. Cohen's *d*, Pearson's *r*), indicating how they were calculated

Our web collection on statistics for biologists contains articles on many of the points above.

Software and code

Policy information about availability of computer code

Data collection	Behavior data was collected in Noldus Ethovision XT 15 software, for the fear conditioning tests the appartus and software from VideoFreeze™ Video Fear Conditioning Software from Med Associates was used.
Data analysis	All the data was analyzed using python 3.11, and statistics for behavioral test was calculated using GraphPad Prism 10 software. Images were analysed using the Olympus OlyVIA3.4.1, Qupath 0.4.4. and Fiji 1.54f softwares with straighten plugin.

For manuscripts utilizing custom algorithms or software that are central to the research but not yet described in published literature, software must be made available to editors and reviewers. We strongly encourage code deposition in a community repository (e.g. GitHub). See the Nature Portfolio guidelines for submitting code & software for further information.

Data

Policy information about availability of data

All manuscripts must include a data availability statement. This statement should provide the following information, where applicable:

- Accession codes, unique identifiers, or web links for publicly available datasets
- A description of any restrictions on data availability
- For clinical datasets or third party data, please ensure that the statement adheres to our policy

Accession Code: PRJNA1150339
DOI: https://dataview.ncbi.nlm.nih.gov/object/PRJNA1150339?reviewer=rj6u513ttc2krsdi71jcvhpi2u

Research involving human participants, their data, or biological material

Policy information about studies with human participants or human data. See also policy information about sex, gender (identity/presentation), and sexual orientation and race, ethnicity and racism.

Reporting on sex and gender	*Use the terms sex (biological attribute) and gender (shaped by social and cultural circumstances) carefully in order to avoid confusing both terms. Indicate if findings apply to only one sex or gender; describe whether sex and gender were considered in study design; whether sex and/or gender was determined based on self-reporting or assigned and methods used. Provide in the source data disaggregated sex and gender data, where this information has been collected, and if consent has been obtained for sharing of individual-level data; provide overall numbers in this Reporting Summary. Please state if this information has not been collected. Report sex- and gender-based analyses where performed, justify reasons for lack of sex- and gender-based analysis.*
Reporting on race, ethnicity, or other socially relevant groupings	*Please specify the socially constructed or socially relevant categorization variable(s) used in your manuscript and explain why they were used. Please note that such variables should not be used as proxies for other socially constructed/relevant variables (for example, race or ethnicity should not be used as a proxy for socioeconomic status). Provide clear definitions of the relevant terms used, how they were provided (by the participants/respondents, the researchers, or third parties), and the method(s) used to classify people into the different categories (e.g. self-report, census or administrative data, social media data, etc.) Please provide details about how you controlled for confounding variables in your analyses.*
Population characteristics	*Describe the covariate-relevant population characteristics of the human research participants (e.g. age, genotypic information, past and current diagnosis and treatment categories). If you filled out the behavioural & social sciences study design questions and have nothing to add here, write "See above."*
Recruitment	*Describe how participants were recruited. Outline any potential self-selection bias or other biases that may be present and how these are likely to impact results.*
Ethics oversight	*Identify the organization(s) that approved the study protocol.*

Note that full information on the approval of the study protocol must also be provided in the manuscript.

Field-specific reporting

Please select the one below that is the best fit for your research. If you are not sure, read the appropriate sections before making your selection.

☒ Life sciences ☐ Behavioural & social sciences ☐ Ecological, evolutionary & environmental sciences

For a reference copy of the document with all sections, see nature.com/documents/nr-reporting-summary-flat.pdf

Life sciences study design

All studies must disclose on these points even when the disclosure is negative.

Sample size	For the whole body KO animals ,we estimated number of animals based on previous mouse WT behavioral studies. For the floxed animals we estimated the numbers based on what the wholebody KO data demonstrated after initial analysis.
Data exclusions	No data was excluded from analyses.
Replication	For all the mice experiments showing in situ hybridizations and immunostainings atleast 3 biological replicates were used. For all the graphical representations of the data, the number of samples used are mentioned in their respective figure legends. All mouse experiments performed in this manuscript with 3 biological replicates with the following exceptions: For the behavior experiments, number of animals used are provided in the figure legend and methods for each test. For the tracing experiments of the whole body KO animals n = 5 (WT), 5 (Het), 4 (KO), and conditional knockouts the numbers n = 4 (WT), 5 (cHet) and 4 (cKO , that are also are provided in figure legends. Human, macaque and Chicken in situ hybridization experiments utilized 1 biological and 2 technical replicates. For the expression analysis of Tfap2d in human, macaque and chicken we used publicly available datasets, the references for which are provided in the text, legends and methods.<br>All experimental findings were reproducible.
Randomization	While randomization wasn't relevant for the primary mutant vs. control comparison, other aspects of the study required careful design to minimize bias. For behavioral analysis, each cohort consisted of littermates (WT, HET, KO) housed together, to avoid confounding housing effects on statistical analyses. The experimental cohort comprised of male and female littermates aged between PD 120-180. For histological analysis, littermates were used to minimize genetic variation unrelated to the target gene. To reduce technical variation, littermate samples were processed in parallel for immunostaining and in situ hybridization using the same batches of reagents and identical imaging conditions. Including samples from multiple litters further enhanced reproducibility.
Blinding	Data collection was performed by independent investigators. For all the experiments were performed on the littermates that had different genotypes like WT, HET and KO. For the tracing experiments equal in amount of the tracer was injected using stereotaxic coordinates. Irrespective of the genotype, only those brains for analysis were selected that displayed right targeting of mPFC. The behavior data was acquired blind of the genotype and the output directly from the software was used to calculate the statistics. In this specific set of histology and in situ experiments, blinding was not possible because it was necessary to know the genotype of each sample to ensure that sufficient

numbers of each genotype (WT, HET, KO) were included in the subsequent analyses. This approach was essential to maintain a balanced experimental design and ensure the statistical validity of the results. Further, these experiments were repeated with different cohorts to ensure reproducibility.

Reporting for specific materials, systems and methods

We require information from authors about some types of materials, experimental systems and methods used in many studies. Here, indicate whether each material, system or method listed is relevant to your study. If you are not sure if a list item applies to your research, read the appropriate section before selecting a response.

Materials & experimental systems

n/a	Involved in the study
☐	☒ Antibodies
☐	☒ Eukaryotic cell lines
☒	☐ Palaeontology and archaeology
☐	☒ Animals and other organisms
☒	☐ Clinical data
☒	☐ Dual use research of concern
☒	☐ Plants

Methods

n/a	Involved in the study
☒	☐ ChIP-seq
☒	☐ Flow cytometry
☐	☒ MRI-based neuroimaging

Antibodies

Antibodies used

SATB1 (1:500, Santacruz, sc-5989, Balamotis et al, PMID: 22064485,) ,
anti-TBR1 (1:200, Abcam, Cat. ab31940, Rabbit polyclonal IgG, IHC; Han et al. 2011 (PMID: 21285371))
anti-NR4A2 (1:500, R&D systems, Cat. AF2156, Polyclonal Goat IgG, IHC, Shibata et al. 2021 (PMID: 34599305))
anti-CUX1 (1:500, Santa Cruz Biotechnology, Cat. sc-13024,rabbit polyclonal IgG, IHC, Kaur etal, PMID: 32707082)
BCL11B (1:2000, Abcam, ab18465; rat monoclonal IgG [25B6], IHC, Kaur etal, PMID: 32707082)
FOS(1:1000, Cell Signalling, 2250, Mineur et al PMID: 17320916)
anti-GFAP (1:2000, Sigma-Aldrich, Cat. G3893,mouse monoclonal IgG1 (G-A-5), IHC, Tebbencamp et al. 2018 (PMID: 30318146)).
GFP (1:500; Abcam, ab13970; Chicken polyclonal IgY, IHC, Kaur etal, PMID: 32707082)
RFP (1:500, Abcam, ab124754; Kaur etal, PMID: 32707082)
anti-cleaved capase3 (1:500; Cell Signaling, Cat. 9611, rabbit polyclonal IgG, IHC, Shim et al. 2012 (PMID: 22678282))
anti-SOX11 (Abcam, ab229185, 1080158-1),
PolII (Sigma-Aldrich, 05-623),IgG (Diagenode, C15410206)
ADGRE1 (1:500, Biorad, MCA497RT, Shi et al., PMID: 31197172),
Alexa Fluor® 594 AffiniPure Donkey Anti-Goat IgG,1:250- 1:500,Jackson ImmunoResearch, Catalog No. 705-585-147, Lot No.169491
Alexa Fluor® 594 AffiniPure Donkey Anti-Rabbit IgG, 1:250- 1:500, Jackson ImmunoResearch, Catalog No.711-585-152, Lot No.171656
Alexa Fluor® 647 AffiniPure Donkey Anti-Mouse IgG, 1:250- 1:500, Jackson ImmunoResearch, Catalog No.715-605-151, Lot No.168259
Alexa Fluor® 594 AffiniPure Donkey Anti-Mouse IgG, 1:250- 1:500, Jackson ImmunoResearch, Catalog No.715-585-150, Lot No.167435
Alexa Fluor® 488 AffiniPure Donkey Anti-Chicken IgY, 1:250- 1:500, Jackson ImmunoResearch, Catalog No.703-545-155, Lot No.168728
Alexa Fluor® 594 AffiniPure Donkey Anti-Goat IgG (H+L), 1:250- 1:500, Jackson ImmunoResearch, Catalog No. 705-585-003
Alexa Fluor® 647 AffiniPure™ Donkey Anti-Goat IgG (H+L), 1:250- 1:500, Jackson ImmunoResearch, Catalog No.705-605-003
Donkey anti mouse - 594, 1:250- 1:500, Jackson ImmunoResearch, Catalog No.715-585-150
Alexa Fluor® 594 AffiniPure™ Goat Anti-Rabbit IgG (H+L), 1:250- 1:500, Jackson Immunoresearch, Catalog No.111-585-003
Alexa Fluor® 647 AffiniPure™ Donkey Anti-Goat IgG (H+L), 1:250- 1:500, Jackson ImmunoResearch, Catalog No.705-605-003, Lot No.705605003

Validation

SOX11 antibody was validated using the SOX11 KO mice (Figure 1e, Extended data figure 2)

Eukaryotic cell lines

Policy information about cell lines and Sex and Gender in Research

Cell line source(s)

Neuro2a mouse neuroblastoma cell line was purchased from ATCC.

Authentication

The cell line was authenticated by morphology or genotyping, and no commonly misidentified lines were used

Mycoplasma contamination

The lines tested negative for mycoplasma contamination, checked monthly using the MycoAlert Mycoplasma Detection Kit (Lonza).

Commonly misidentified lines
(See ICLAC register)

No commonly misidentified lines were used .

Animals and other research organisms

Policy information about studies involving animals; ARRIVE guidelines recommended for reporting animal research, and Sex and Gender in Research

Laboratory animals	B6SJLF1/J (The Jackson Laboratory) were used for the Tfap2d wholebody and cKO animals. The SOX11 and SOX4 cKO lines were mixed breed. Mentioned in methods under animals. Monkey aging post conception day 105. human aging post conception week 1 and chicken samples collected from E17 were used in this study.
Wild animals	The study did not involve wild animals.
Reporting on sex	Animals were chosen unbaised to sex in all analysis.
Field-collected samples	No field samples were collected
Ethics oversight	All the protocols were approved by Yale IACUC.

Note that full information on the approval of the study protocol must also be provided in the manuscript.

Plants

Seed stocks	*Report on the source of all seed stocks or other plant material used. If applicable, state the seed stock centre and catalogue number. If plant specimens were collected from the field, describe the collection location, date and sampling procedures.*
Novel plant genotypes	*Describe the methods by which all novel plant genotypes were produced. This includes those generated by transgenic approaches, gene editing, chemical/radiation-based mutagenesis and hybridization. For transgenic lines, describe the transformation method, the number of independent lines analyzed and the generation upon which experiments were performed. For gene-edited lines, describe the editor used, the endogenous sequence targeted for editing, the targeting guide RNA sequence (if applicable) and how the editor was applied.*
Authentication	*Describe any authentication procedures for each seed stock used or novel genotype generated. Describe any experiments used to assess the effect of a mutation and, where applicable, how potential secondary effects (e.g. second site T-DNA insertions, mosiacism, off-target gene editing) were examined.*

Magnetic resonance imaging

Experimental design

Design type	Ex vivo whole brains of wildtype controls, Tfap2d HET and TFAP2D KO mutant mice were imaged and analyzed
Design specifications	N/A
Behavioral performance measures	N/A

Acquisition

Imaging type(s)	Diffusion tensor imaging
Field strength	9.4T
Sequence & imaging parameters	3D echo-planner-imaging (EPI) diffusion sequence (b=1500s/mm2; 30 directions) with the following parameters57: repetition time=1250ms; echo time=26ms. The resolution was 0.1mm isotropic. Overall scanning time was 19.5h.
Area of acquisition	Whole brain scan

Diffusion MRI ☒ Used ☐ Not used

Parameters: *Specify # of directions, b-values, whether single shell or multi-shell, and if cardiac gating was used.*

Preprocessing

Preprocessing software	Preprocessing was done with MRtrix361 software
Normalization	Data were not normalized since there are no normalization atlases for a specific KO group used in the study.
Normalization template	N/A
Noise and artifact removal	Denoising was done using the MRtrix denoise algorithm, and bias field corrections were done using corresponding ANTs'

Noise and artifact removal | algorithm. Motion parameters and physiological signals were non-existed since the MRI was done ex-vivo.

Volume censoring | N/A

Statistical modeling & inference

Model type and settings | N/A

Effect(s) tested | Number of streamlines between the two groups

Specify type of analysis: ☐ Whole brain ☒ ROI-based ☐ Both

Anatomical location(s) | *Describe how anatomical locations were determined (e.g. specify whether automated labeling algorithms or probabilistic atlases were used).*

Statistic type for inference (See Eklund et al. 2016) | Two-tailed paired t-test or two tailed Mann-Whitney test

Correction | N/A

Models & analysis

n/a	Involved in the study
☐	☒ Functional and/or effective connectivity
☒	☐ Graph analysis
☒	☐ Multivariate modeling or predictive analysis

Functional and/or effective connectivity | Structural connectivity between brain regions was determined by the number of streamlines derived by seeding streamlines from a specific region of interest with an ending point in another region of interest.
